# Supplementary material for: Human astrocytes and microglia show augmented ingestion of synapses in Alzheimer’s disease via MFG-E8
Source: Cell Rep Med. 2023 Aug 30;4(9):101175. doi: 10.1016/j.xcrm.2023.101175 (PMC10518633; doi:10.1016/j.xcrm.2023.101175)
Supplement: Document S1. Figures S1–S6, Tables S1 and S2 [file mmc1.pdf]

Cell Reports Medicine, Volume *4*

Supplemental information

Human astrocytes and microglia show augmented ingestion of synapses in Alzheimer's disease via MFG-E8

Makis Tzioras, Michael J.D. Daniels, Caitlin Davies, Paul Baxter, Declan King, Sean McKay, Balazs Varga, Karla Popovic, Madison Hernandez, Anna J. Stevenson, Jack Barrington, Elizabeth Drinkwater, Julia Borella, Rebecca K. Holloway, Jane Tulloch, Jonathan Moss, Clare Latta, Jothy Kandasamy, Drahoslav Sokol, Colin Smith, Veronique E. Miron, Ragnhildur Thóra Káradóttir, Giles E. Hardingham, Christopher M. Henstridge, Paul M. Brennan, Barry W. McColl, and Tara L. Spires-Jones

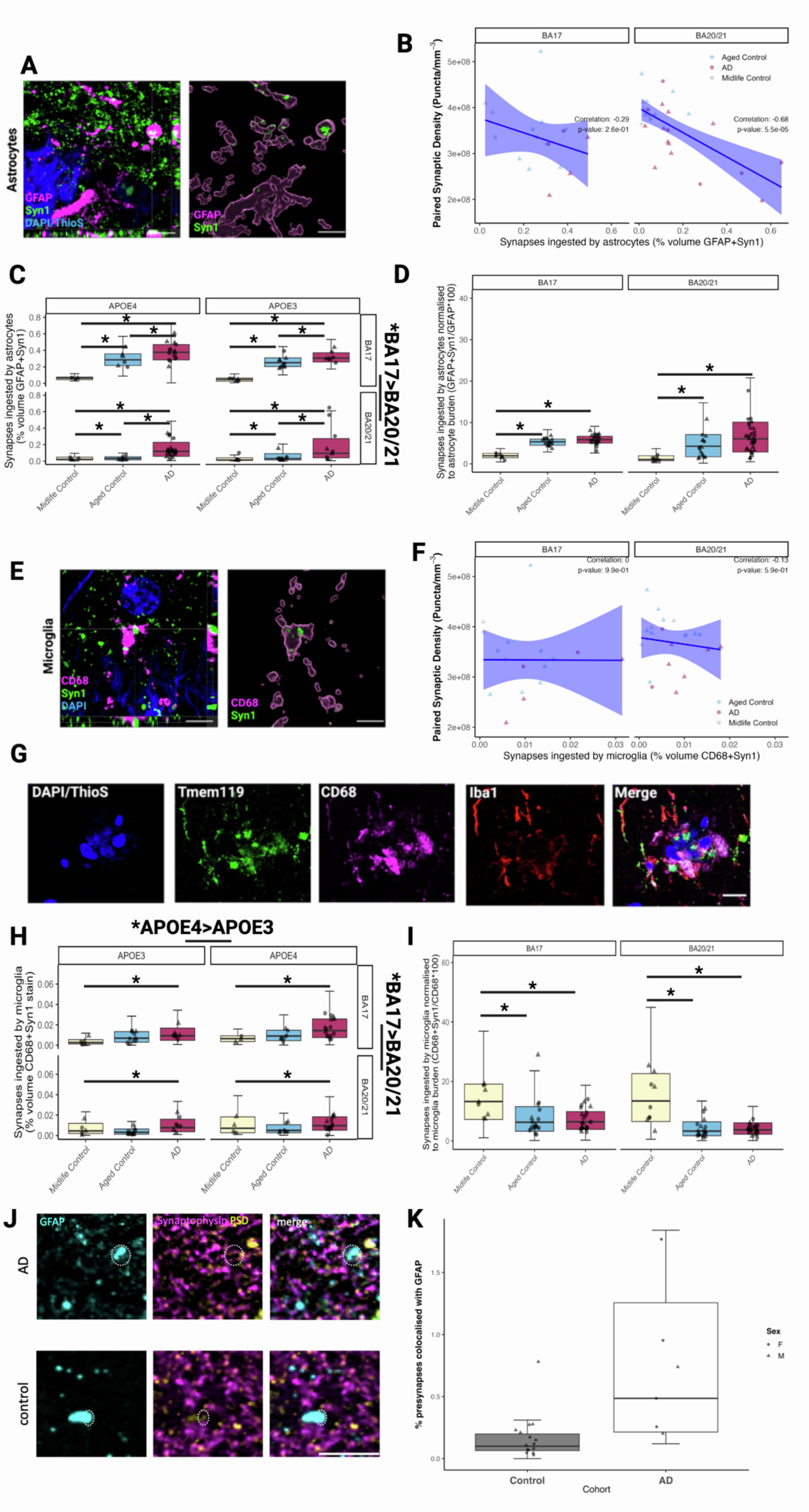

Figure S1. Supplementary statistics and validation microscopy, Related to Figures 1-3. (A) Super-resolution confocal using an Airyscan microscope confirm synaptic engulfment by astrocytes in AD (orthogonal view left , 3D reconstruction right). Scale bar represents 5μm. (B) Correlation analysis shows a significant negative correlation between

synapse density determined with array tomography and synaptic colocalization with astrocytes in BA20/21. (C) There is an effect of *APOE* genotype with *APOE4* carriers having more astrocyte engulfment than *APOE3* carriers. Also there is more engulfment in BA17 compared to BA20/21. (D) When normalized to GFAP burden, synaptic colocalization with GFAP was increased in AD compared to mid-life controls and increased in healthy ageing compared to mid-life controls. (E) Super-resolution confocal using an Airyscan microscope confirm synaptic engulfment by microglia in AD (orthogonal view left , 3D reconstruction right). Scale bar represents 5µm. (F) Correlation analysis shows no significant correlations between synapse density determined with array tomography and synaptic colocalization with microglia in either brain region. (G) CD68 staining was confirmed to colocalise with Tmem119 and Iba1. Scale bar represents 20µm. (H) There is an effect of *APOE* genotype with *APOE4* carriers having more astrocyte engulfment than *APOE3* carriers. Also there is more engulfment in BA17 compared to BA20/21. (I) When normalized to CD68 burden, the synaptic ingestion by microglia was no longer higher in AD cases compared to controls indicating the increase in AD in microglial ingestion of synapses is driven by microgliosis. (J) Array tomography imaging of GFAP and synaptic proteins confirms synaptic ingestion by astrocytes. Scale bar represents 10µm. (K) There is significantly more synaptic ingestion by astrocytes in AD than control when measured with array tomography (ANOVA after linear mixed effects model, $F[1,16]=12.11$, $p=0.003$).

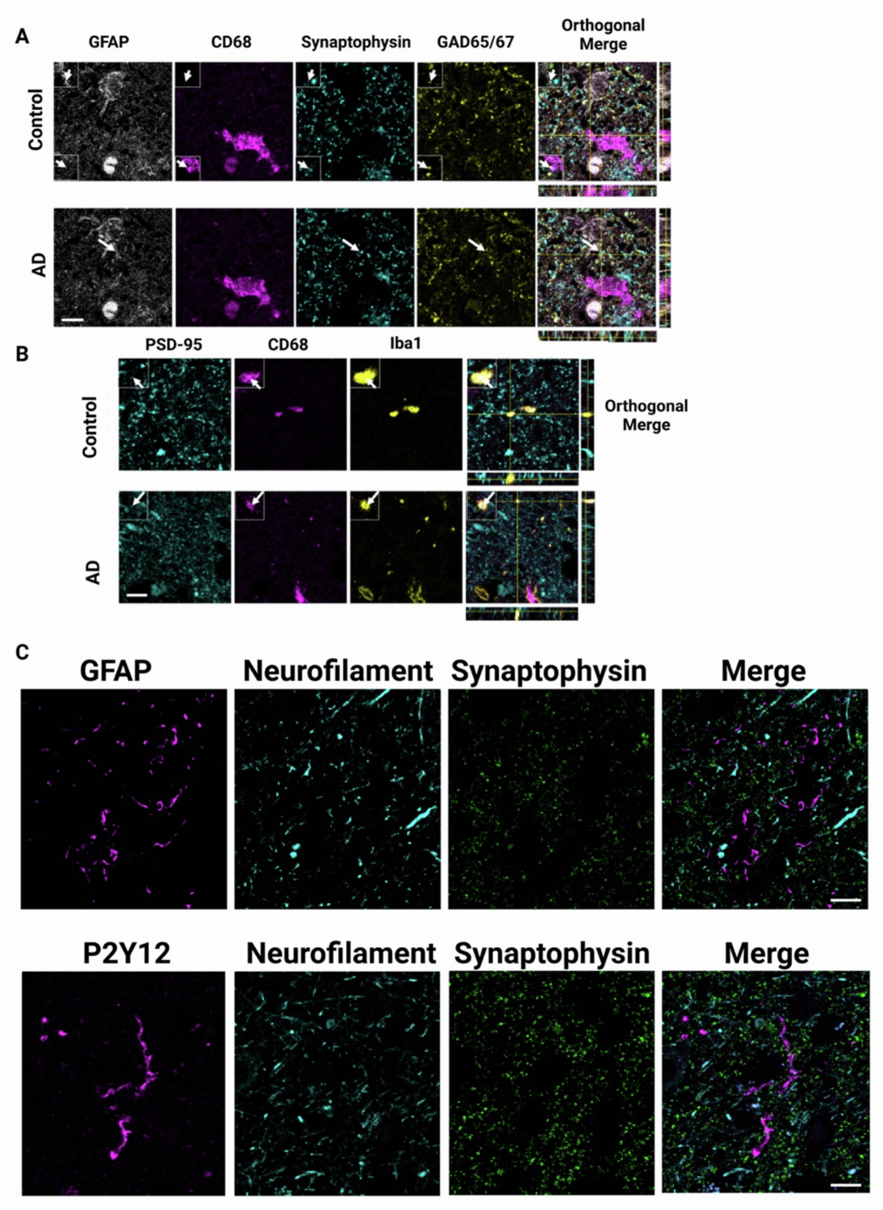

Figure S2. Glial co-staining with multiple synaptic markers. Related to Figures 1-3.
(A) Confocal images of staining with GFAP, CD68, synaptophysin, and GAD65/67 shows inhibitory synaptic protein inside astrocytes and microglia (arrows). (B) Excitatory postsynaptic protein PSD95 is also observed in CD68 and Iba1double positive microglia (arrows, B). (C) Staining with axonal neurofilament (cyan) alongside GFAP or P2Y12 (magenta) and synaptophysin (green) did not show substantial colocalization between astrocytes or microglia and axonal neurofilament. Scale bars represent 10mm. Insets are 5 x 5 mm.

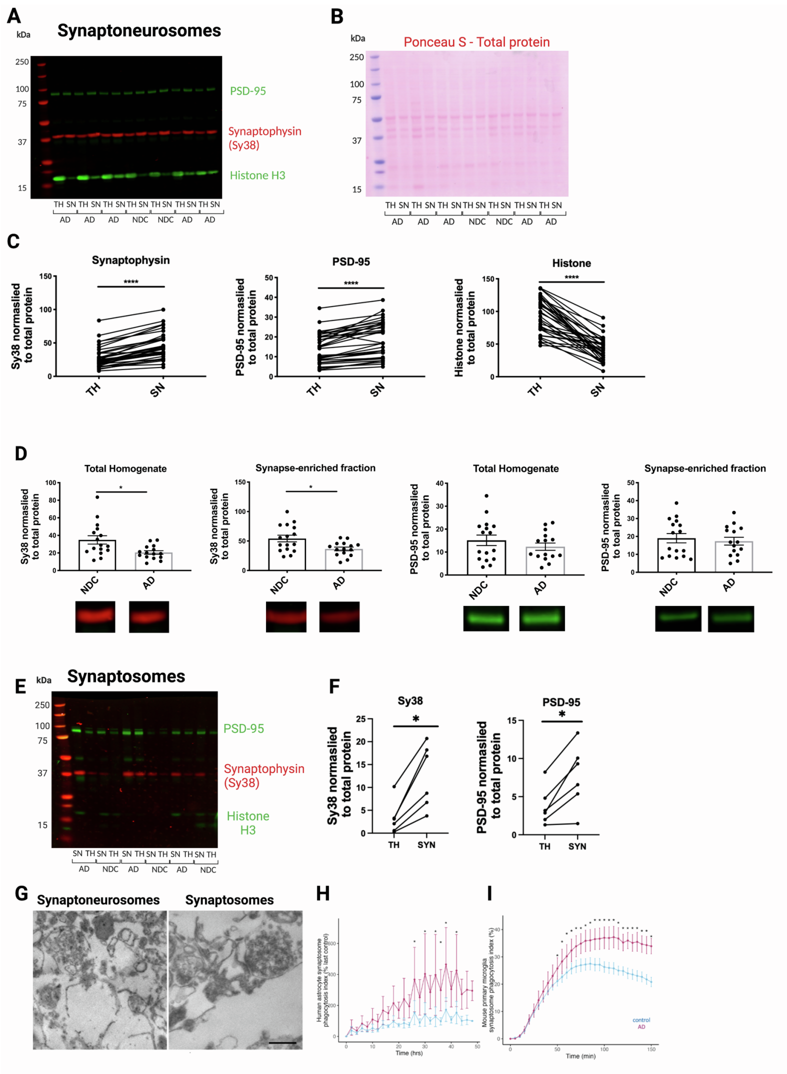

Figure S3. Validation of synaptoneurosome and synaptosome preparations. Related to Figures 4-6. (A) Representative image of full-length Western blot, indicating whether a sample is from total homogenate (TH) or synaptoneurosomes (SN), and their corresponding disease status. Bands were quantified on Image Studio, and normalised to total protein, quantified by Ponceau S. (B) Ponceau S staining for total protein from gel shown in A (NDC=no disease control, AD=Alzheimer's disease). (C) Significantly increased protein levels of the pre- and post-synaptic markers synaptophysin (Sy38) and PSD-95, respectively, as well as decreased protein levels of histone (H3), indicating exclusion of non-synaptic material (Wilcoxon matched-pairs signed rank test, ****$p<0.0001$, n=31). Lines link the two different preparations from the same case. (D) Decreased levels of synaptophysin (Sy38) in the total homogenate (Mann-Whitney test, p=0.0106) and synaptoneurosome fraction (unpaired Student's t-test, p=0.0121) of AD cases (n=15), compared to age-matched NDC cases (n=16), as detected by Western blot. PSD-95 protein levels were not different between AD and NDC groups in total homogenate (unpaired Student's t-test, p=0.332), nor in the synaptoneurosome fraction (unpaired Student's t-test, p=0.627). (E) Representative image of full-length Western blot for synaptophysin, PSD-95 and histone H3, indicating whether a sample is from total homogenate (TH) or synaptoneurosomes (SN), and their corresponding disease status. Bands were quantified on Image Studio, and normalised to total protein, quantified by Ponceau S. (F) Significantly increased protein levels of the pre- and post-synaptic markers synaptophysin (Sy38) and PSD-95, respectively, (Wilcoxon matched-pairs signed rank test, *$p<0.05$, n=3). Lines link the two different preparations from the same case. (G) Synaptoneurosome and synaptosome pellets were embedded for electron microscopy confirming synaptic structures in both types of synaptic enrichment (scale bar 300 nm). (H) As seen with synaptoneurosomes, pHrodo tagged human synaptsomes from AD brain were phagocytosed more and faster by human astrocytes. (I) AD-derived human synapotosomes also ingested more than control by mouse microglia, similar to the synaptoneurosomes. For statistics, *$p<0.05$.

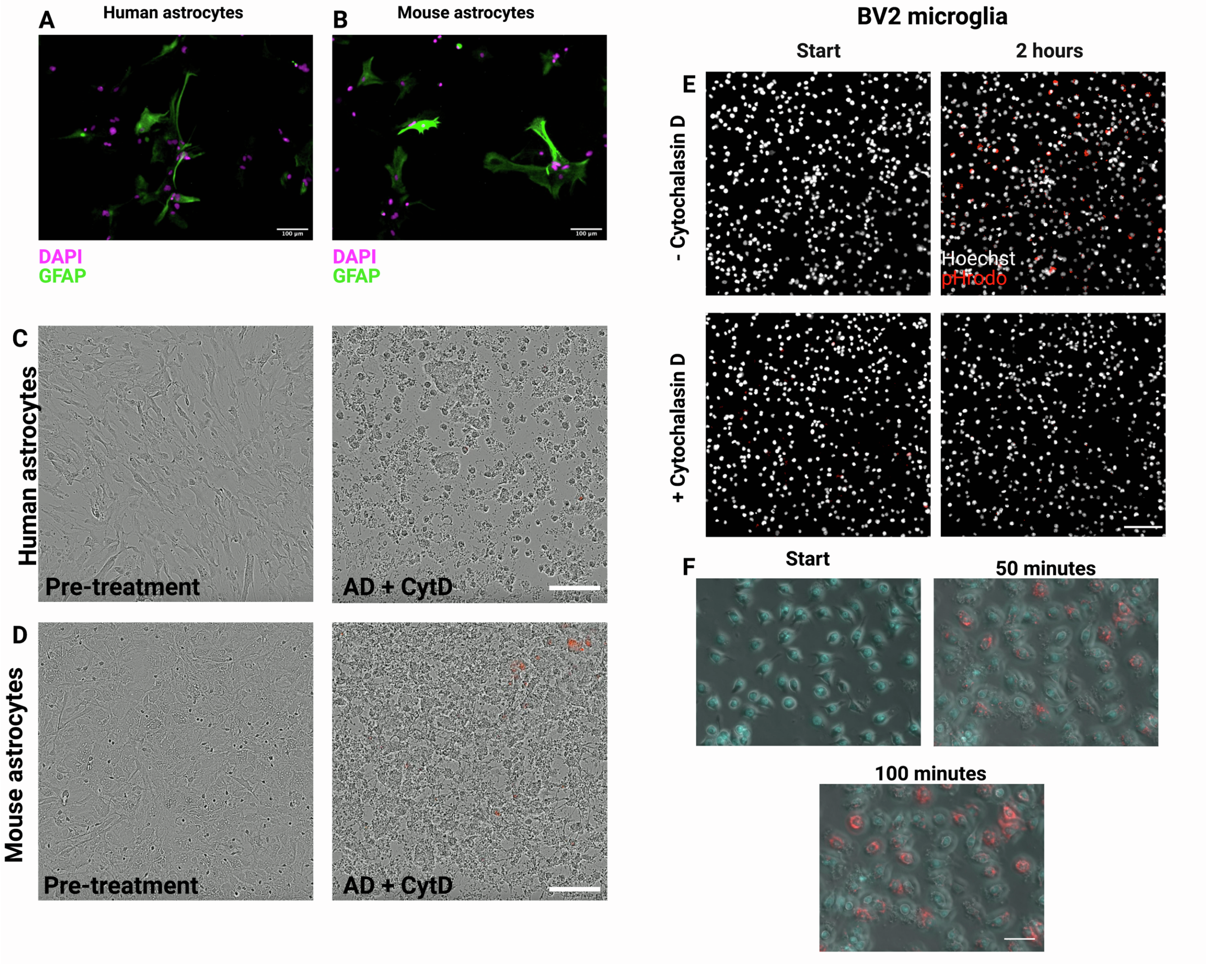

Figure S4. Validation of synaptoneurosome ingestion by astrocytes and microglia. Related to Figures 4-6. (A) Primary human astrocytes stained for GFAP (green) shows most cells express the marker, indicating a pure astrocytic culture. (B) Primary mouse astrocytes stained for GFAP (green) shows cells express the marker, although some cells do not. Scale bars represent 100µm. (C) Primary human astrocytes treated with cytochalasin D prior to phagocytosis assay, blocking phagocytosis. Scale bar 200µm. (D) Primary mouse astrocytes treated with cytochalasin D prior to phagocytosis assay, blocking phagocytosis. Scale bar 200µm. (E) Still images from live imaging of BV2 microglia (Hoechst-positive nuclei in grey) undergoing phagocytosis of human synaptoneurosomes tagged with pHrodo. Synaptoneurosomes can be seen in red as they enter the acidic phago-lysosomal compartment of the cell. (F) Cells treated with 10µM of Cytochalasin D 30 minutes prior to the experiment showed no phagocytosis. Scale bar represents 30µm. (G) Live imaging of BV2 microglia (phase with Hoechst-positive nuclei in cyan) undergoing phagocytosis of human synaptoneurosomes tagged with pHrodo (can be seen as small spheroids on phase contrast). Synaptoneurosomes become red once they enter the acidic phago-lysosomal compartment of the cell. Each panel represents an image 50 minutes apart. Scale bar represents 50µm.

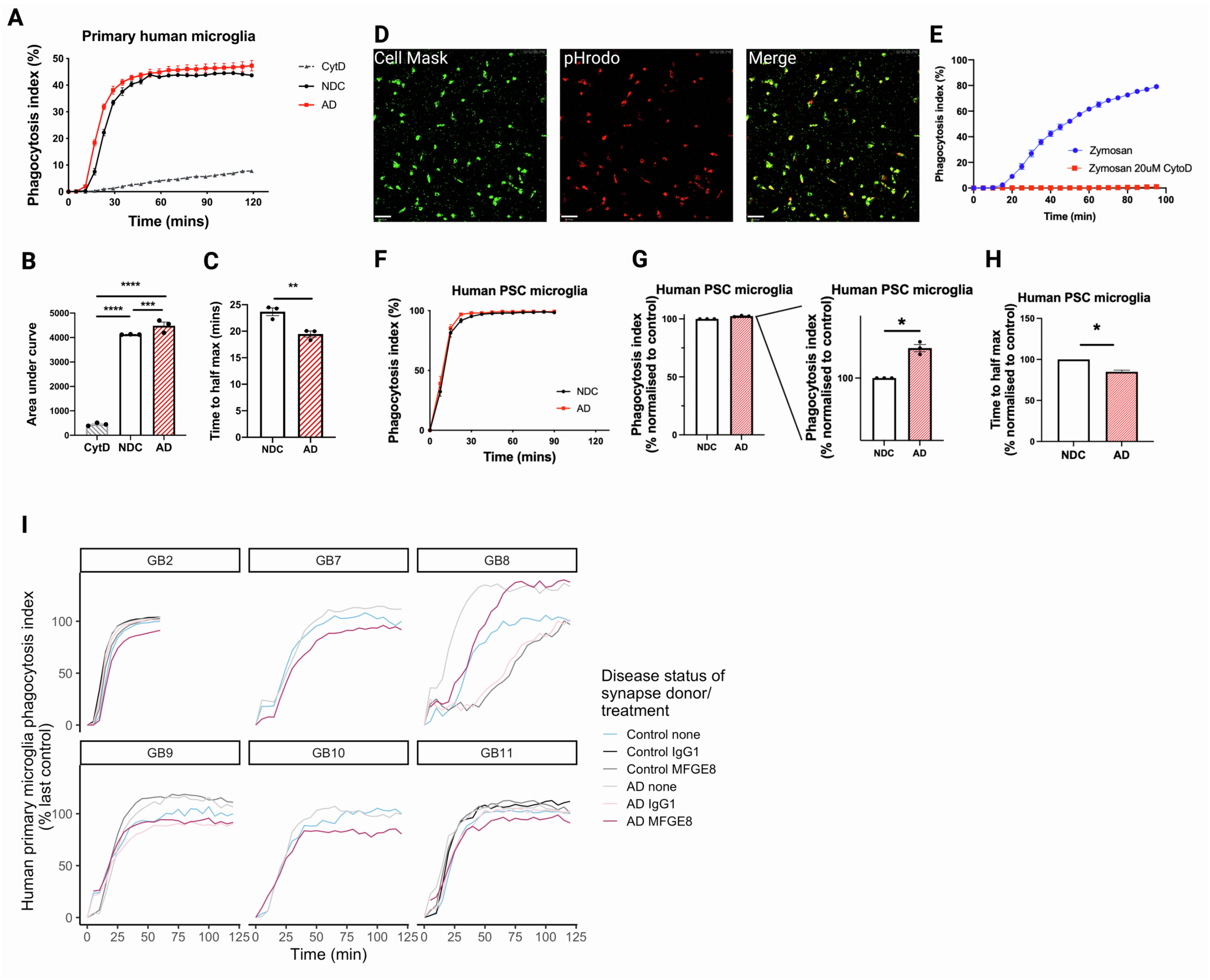

Figure S5. Increased phagocytosis of AD-derived synaptoneurosomes by human iPSC and primary microglia. Related to Figures 5 and 6. (A) Phagocytosis index of primary human microglia from an epilepsy case engulfing human synaptoneurosomes (n=1 human sample, replicated in 3 wells from an average of 9 images per well). CytD= cytochalasin D, NDC= non-demented control, AD= Alzheimer's disease. (B) Area under curve from A (one-way ANOVA with Tukey's multiple comparisons test, p=0.0004). (C) Time to half-maximum phagocytosis was calculated from the curve shown in A (unpaired Student's t-test, p=0.0098). For statistics, **p<0.01, ***p<0.001, ****p<0.0001. Data shown as mean ± SEM. (D) Representative images of human PSC derived microglia-like cells labelled with Cellmask Deep red (green), showing phagocytosed pHrodo labelled synaptoneurosomes (red) at the end of imaging time. Scalebar: 80µm. (E) Zymosan beads are readily phagocytosed by human PSC microglia in culture, and treatment of cytochalasin D (CytoD) is sufficient to completely abolish it. Data shown as mean ± SEM. (F) All cell lines showed phagocytosis of SNS particles, the responsiveness and dynamics of microglia to AD and control material uptake depends on the individual cell lines. (G) Total amount of labelled particles as measured by the area under the curve was increased in PSC derived microglia presented with synaptoneurosomes from AD brain compared to NDC (one sample t-test, p=0.0142, hypothetical value=100). (H) PSC derived microglia-like cells phagocytosed AD SNS particles faster compared to NDC particles measured by the half max time (one sample t-test, p=0.016, hypothetical value=100). In all conditions, n=3 indicating lines from separate iPSC donors. For statistics, *p<0.05, and data shown as mean ± SEM). CytD= cytochalasin D, NDC= no disease control, AD= Alzheimer's disease. (I) The small surgical samples from which microglia were derived were not always sufficient to test all conditions. From our 11 donors, all had AD vs control synapse conditions, two had these and in addition AD synapses treated with MFG-E8 antibody, and 4 included all conditions (control and AD synapses with anti-MFG-E8, IgG1 control, or no treatment). In all 6 cases with MFG-E8 antibody pre-incubation, MFG-E8 antibody pre-treatment reduced AD synaptic phagocytosis compared to AD without antibody pre-treatment. In the 4 cases with IgG control, 2 had clear reduction in phagocytosis with MFGE8 compared to control IgG (GB11, GB2), one had no difference between MFGE8 and IgG (GB9), and one had increased phagocytosis with MFGE8 compared to IgG (GB8). From these data we conclude that while there is not a clear rescue of phenotype specifically with the MFG-E8 antibody treatment as seen for astrocytes, the microglial data show "responders" and "non-responders" to this treatment compared to IgG1 and all cases had a clear reduction in phagocytosis with either IgG1 or MFGE-8 treatment indicating that IgG on synapses may be an opsonin recognized by microglia .

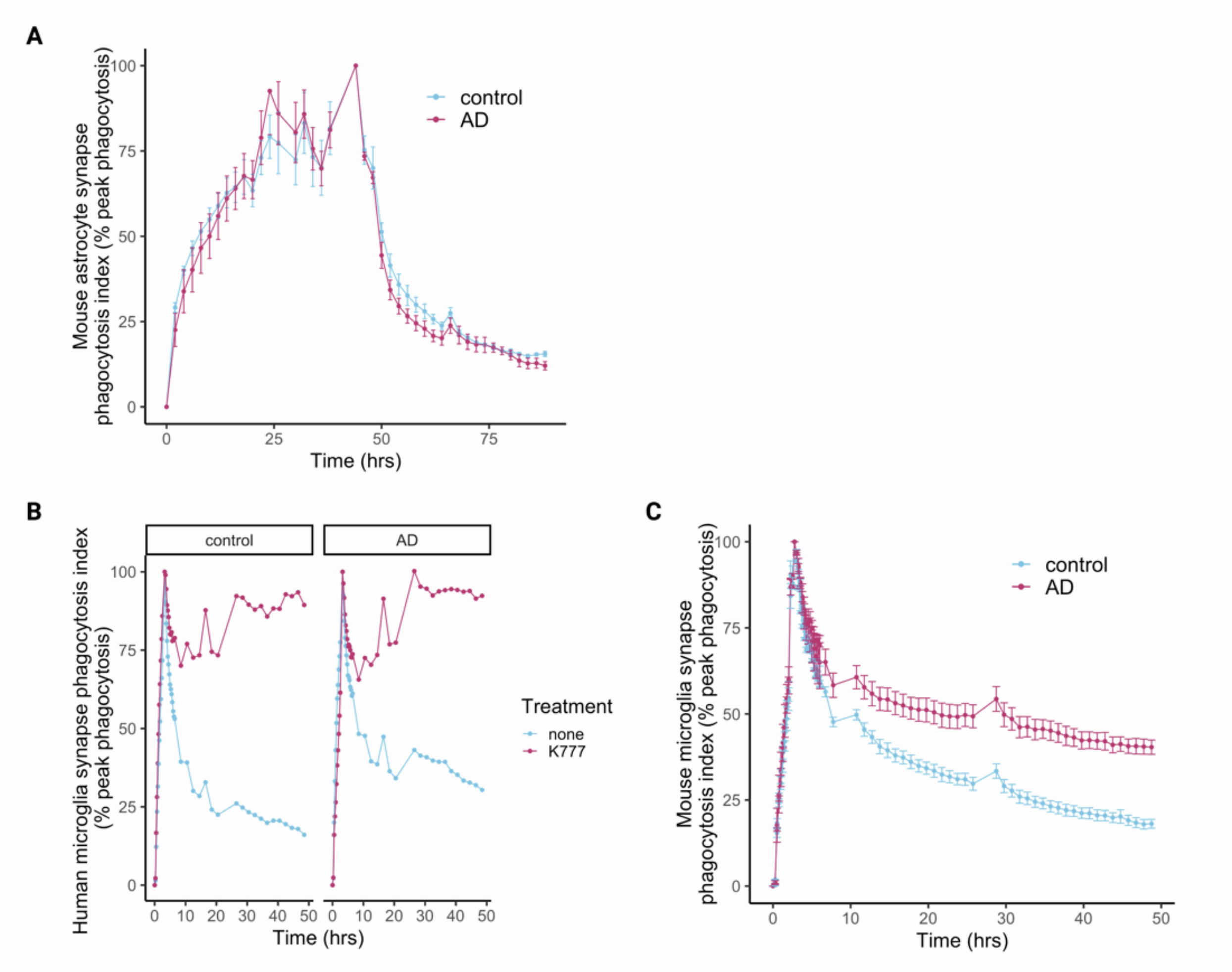

Figure S6. Degradation assays of mouse and human primary microglia, Related to Figures 5 and 6. (A) Degradation assay in primary mouse astrocytes (n=4 replicates) of human control and Alzheimer's disease (AD) synaptoneurosomes shows no differences in degradation throughout the assay. (B) Degradation assay in primary human microglia (n=1, GB12) of human control and Alzheimer's disease (AD) synaptoneurosomes shows no differences in degradation in the 2 hours of the assay. Both control and AD synaptoneurosomes are efficiently degraded over time, which is blocked by the pan-cathepsin inhibitor K777. (C) Degradation assay in primary mouse microglia (n=4 adult mice) of human control and Alzheimer's disease (AD) synaptoneurosomes shows no differences in degradation in the first 2 hours of the assay. Both control and AD synaptoneurosomes are efficiently degraded over time.

BBN/ CaseID	SD number	Disease	APOE	Age (years)	Gender	PMI (hrs)	Braak Stage	Experiment
29693	SD002/17	Midlife control	APOE4	49	F	94	NA	IHC
24479	SD005/15	Midlife control	APOE4	46	F	76	NA	IHC
33613	SD014/18	Midlife control	APOE3	46	F	99	NA	IHC
28959	SD022/16	Midlife control	APOE3	39	M	86	NA	IHC
30169	SD022/17	Midlife control	APOE4	48	M	58	NA	IHC
28960	SD026/16	Midlife control	APOE3	37	F	126	NA	IHC
34244	SD036/18	Midlife control	APOE3	49	F	69	NA	IHC
24342	SD053/14	Midlife control	APOE3	33	M	47	NA	IHC
29906	SD013/17	Midlife control	APOE4	51	M	52	NA	IHC
1.28793	SD017/16	Aged Control	APOE3	79	F	72	2	IHC
14395	SD014/13	Aged Control	APOE3	74	F	41	NA	IHC + SNS
19686	SD063/13	Aged Control	APOE3	76	F	75	1	IHC + SNS
20122	SD003/14	Aged Control	APOE3	59	M	74	NA	IHC + SNS
22612	SD022/14	Aged Control	APOE3	61	M	70	NA	IHC + SNS
26495	SD024/15	Aged Control	APOE3	78	M	39	1	IHC + SNS
28402	SD051/15	Aged Control	APOE3	78	M	49	1	IHC + SNS
28406	SD001/16	Aged Control	APOE3	79	M	72	2	IHC + SNS
28797	SD025/16	Aged Control	APOE3	79	M	57	NA	IHC + SNS
29086	SD034/16	Aged Control	APOE3	79	F	68	NA	IHC + SNS
32577	SD002/18	Aged Control	APOE3	81	M	74	2	IHC
1.34131	SD029/18	Aged Control	APOE4	82	M	95	4	IHC
15809	SD029/13	Aged Control	APOE4	58	M	90	NA	IHC + SNS
16425	SD032/13	Aged Control	APOE4	61	M	99	NA	IHC + SNS
20593	SD006/14	Aged Control	APOE4	60	M	52	NA	IHC + SNS
22629	SD035/14	Aged Control	APOE4	59	F	53	NA	IHC + SNS
29082	SD031/16	Aged Control	APOE4	79	F	80	3	IHC + SNS
31495	SD043/17	Aged Control	APOE4	81	M	38	6	IHC + SNS
1.29081	SD030/16	AD	APOE3	90	F	110	2	IHC
1.30142	SD020/17	AD	APOE3	88	F	112	2	IHC
15258	SD026/13	AD	APOE3	65	M	80	6	IHC + SNS
19595	SD062/13	AD	APOE3	87	M	58	6	IHC + SNS
19994	SD002/14	AD	APOE3	87	F	89	6	IHC + SNS
24527	SD056/14	AD	APOE3	81	M	74	5	IHC
28410	SD005/16	AD	APOE3	62	F	109	6	IHC + SNS
28771	SD010/16	AD	APOE3	85	M	91	6	IHC

32929	SD012/18	AD	APOE3	87	F	83	4	IHC
1.265	SD039/15	AD	APOE4	81	M	83	6	IHC
1.26732	SD048/15	AD	APOE4	76	M	66	6	IHC + SNS
1.28796	SD021/16	AD	APOE4	60	F	54	6	IHC
1.29135	SD027/16	AD	APOE4	90	M	73	6	IHC
1.30883	SD034/17	AD	APOE4	61	F	69	6	IHC
1.30973	SD039/17	AD	APOE4	89	F	96	6	IHC
1.31499	SD044/17	AD	APOE4	85	M	78	6	IHC
1.33636	SD017/18	AD	APOE4	93	M	43	2	IHC
1.33698	SD022/18	AD	APOE4	90	F	76	NA	IHC
10591	SD003/13	AD	APOE4	76	M	76	6	IHC + SNS
15256	SD034/13	AD	APOE4	60	M	28	5	IHC
15810	SD018/13	AD	APOE4	73	F	96	6	IHC
15811	SD021/13	AD	APOE4	81	F	41	6	IHC
19690	SD064/13	AD	APOE4	57	M	58	6	IHC + SNS
20995	SD019/14	AD	APOE4	60	M	86	6	IHC + SNS
23394	SD038/14	AD	APOE4	88	F	59	5	IHC + SNS
24322	SD049/14	AD	APOE4	80	M	101	6	IHC + SNS
24526	SD055/14	AD	APOE4	79	M	65	6	IHC
24668	SD058/14	AD	APOE4	96	F	61	6	IHC
25739	SD014/15	AD	APOE4	85	F	45	6	IHC + SNS
26718	SD040/15	AD	APOE4	78	M	74	6	IHC + SNS
29521	SD035/16	AD	APOE4	95	M	96	6	IHC + SNS
29695	SD004/17	AD	APOE4	86	M	72	6	IHC + SNS

Table S1. Human cases used in the study. (NA=not available, IHC = immunohistochemistry, SNS = synaptoneurosome)

CaseID	Age	Gender	Brain Region resected
GB1	55	M	parietal lobe
GB2	50	F	temporal lobe
GB3	50	M	frontal lobe
GB5	50	F	parietal lobe
GB6	60	F	parietal lobe
GB7	60	F	frontal lobe
GB8	60	F	frontal lobe
GB9	50	F	frontal lobe
GB10	60	M	frontal lobe
GB11	70	M	frontal lobe
GB12	71	M	parietal-occipital lobe

Table S2. Data from human donors from glioblastoma surgeries, Related to figures 5 and 6.
