# Supplementary material for: Human astrocytes and microglia show augmented ingestion of synapses in Alzheimer’s disease via MFG-E8
Source: Cell Rep Med. 2023 Aug 30;4(9):101175. doi: 10.1016/j.xcrm.2023.101175 (PMC10518633; doi:10.1016/j.xcrm.2023.101175)
Supplement: Document S2. Article plus supplemental information [file mmc6.pdf]

Cell Reports
Medicine

Article

Human astrocytes and microglia show augmented ingestion of synapses in Alzheimer's disease via MFG-E8

Graphical abstract

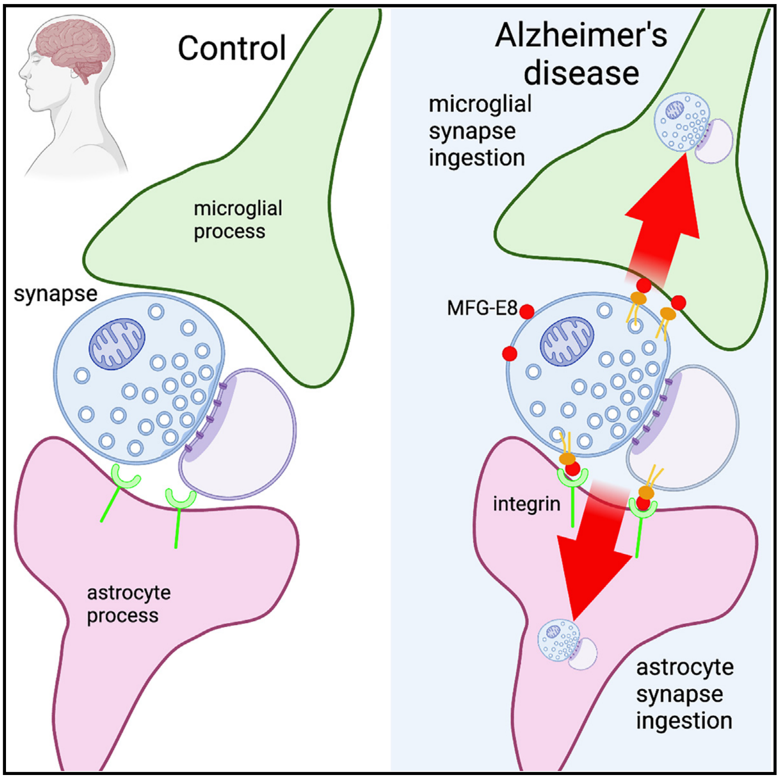

Highlights

- Astrocytes and microglia contain synaptic protein in human postmortem brain tissue
- Synapse ingestion by astrocytes and microglia is increased in Alzheimer's disease
- MFG-E8 is increased on synapses from Alzheimer's brain
- Blocking MFG-E8 reduces synapse phagocytosis by cultured astrocytes and microglia

Authors

Makis Tzioras, Michael J.D. Daniels, Caitlin Davies, ..., Paul M. Brennan, Barry W. McColl, Tara L. Spires-Jones

Correspondence

barry.mccoll@ed.ac.uk (B.W.M.),
tara.spires-jones@ed.ac.uk (T.L.S.-J.)

In brief

Tzioras and colleagues report that synapse ingestion by astrocytes and microglia is increased in Alzheimer's disease brain tissue compared with controls. Human astrocytes and microglia in culture demonstrate enhanced phagocytosis of synapses derived from Alzheimer's brain, which can be reduced to control levels by blocking MFG-E8 protein on the synapses.

Tzioras et al., 2023, Cell Reports Medicine *4*, 101175
September 19, 2023 © 2023 The Author(s).
https://doi.org/10.1016/j.xcrm.2023.101175

CellPress

Cell Reports Medicine

CellPress
OPEN ACCESS

Article

Human astrocytes and microglia show augmented ingestion of synapses in Alzheimer's disease via MFG-E8

Makis Tzioras,[1,2,10] Michael J.D. Daniels,[1,2,10] Caitlin Davies,[1,2] Paul Baxter,[1,2] Declan King,[1,2] Sean McKay,[1,2] Balazs Varga,[3] Karla Popovic,[1,2] Madison Hernandez,[1,2] Anna J. Stevenson,[1,2] Jack Barrington,[1,2] Elizabeth Drinkwater,[1,2] Julia Borella,[1,2] Rebecca K. Holloway,[4] Jane Tulloch,[1,2] Jonathan Moss,[4,5] Clare Latta,[1,2] Jothy Kandasamy,[6] Drahoslav Sokol,[6] Colin Smith,[7] Veronique E. Miron,[1,4,8] Ragnhildur Thóra Káradóttir,[3] Giles E. Hardingham,[1,2] Christopher M. Henstridge,[9] Paul M. Brennan,[6,7] Barry W. McColl,[1,2,11,*] and Tara L. Spires-Jones[1,2,11,12,*]

[1]UK Dementia Research Institute, the University of Edinburgh, Edinburgh EH8 9JZ, UK
[2]Centre for Discovery Brain Sciences, the University of Edinburgh, Edinburgh EH8 9JZ, UK
[3]Wellcome – MRC Cambridge Stem Cell Institute, University of Cambridge, Cambridge CB2 0AW, UK
[4]MRC Centre for Reproductive Health, the University of Edinburgh, Edinburgh EH16 4TJ, UK
[5]The Roslin Institute, the Royal (Dick) School of Veterinary Studies, the University of Edinburgh, Edinburgh EH25 9RG, UK
[6]Department of Clinical Neurosciences, Royal Infirmary of Edinburgh, Edinburgh EH16 4SA, UK
[7]Centre for Clinical Brain Sciences, the University of Edinburgh, Edinburgh EH16 4SB, UK
[8]Barlo Multiple Sclerosis Centre at St. Michael's Hospital, Keenan Research Centre for Biomedical Science, Toronto, ON M5B 1T8, Canada
[9]Division of Systems Medicine, School of Medicine, University of Dundee, Dundee DD1 9SY, UK
[10]These authors contributed equally
[11]These authors contributed equally
[12]Lead contact
*Correspondence: barry.mccoll@ed.ac.uk (B.W.M.), tara.spires-jones@ed.ac.uk (T.L.S.-J.)
https://doi.org/10.1016/j.xcrm.2023.101175

SUMMARY

Synapse loss correlates with cognitive decline in Alzheimer's disease (AD). Data from mouse models suggests microglia are important for synapse degeneration, but direct human evidence for any glial involvement in synapse removal in human AD remains to be established. Here we observe astrocytes and microglia from human brains contain greater amounts of synaptic protein in AD compared with non-disease controls, and that proximity to amyloid-β plaques and the *APOE4* risk gene exacerbate this effect. In culture, mouse and human astrocytes and primary mouse and human microglia phagocytose AD patient-derived synapses more than synapses from controls. Inhibiting interactions of MFG-E8 rescues the elevated engulfment of AD synapses by astrocytes and microglia without affecting control synapse uptake. Thus, AD promotes increased synapse ingestion by human glial cells at least in part via an MFG-E8 opsonophagocytic mechanism with potential for targeted therapeutic manipulation.

INTRODUCTION

Alzheimer's disease (AD) is a neurodegenerative disease characterized by progressive cognitive decline and the accumulation of two pathological protein aggregates in the brain, amyloid-β (Aβ) plaques and phosphorylated tau tangles.[1,2] Oligomeric forms of Aβ and tau are toxic to synapses and contribute to synapse loss.[3–6] In turn, synapse loss is the strongest pathological correlate of cognitive decline in AD[7,8]; however, no therapies have been developed to effectively halt the degeneration and loss of synapses in humans. Controlled synapse elimination is an important aspect of the developing brain and this process is aided by glial cells in the brain, specifically astrocytes and microglia.[9–13] This controlled synaptic refinement, or pruning, relies on multiple pathways, including the complement system,[10] interleukin (IL)-33,[14] and epigenetic factors.[15] Interestingly, it is hypothesized based on data from animal models that glial cells re-activate pathways involved in developmental synaptic pruning in a pathological manner during AD. For instance, in animal models of AD, microglia and astrocytes engulf more synapses both in response to Aβ pathology[16–19] and tau.[20,21] The upregulated synaptic engulfment is often accompanied by cognitive deficits in mice, and importantly, when synaptic engulfment is blocked, there is a rescue of this cognitive deficit.[16,17,22] This suggests that not all synapses that are removed by microglia are degenerating, and highlights an important therapeutic window for rescuing healthy synapses from being lost. There is a paucity of human studies investigating microglial and astrocyte interactions with synapses in disease, which may in part contribute to the lack of effective drugs in trial or approved for AD.[23]

This is an open access article under the CC BY license (http://creativecommons.org/licenses/by/4.0/).

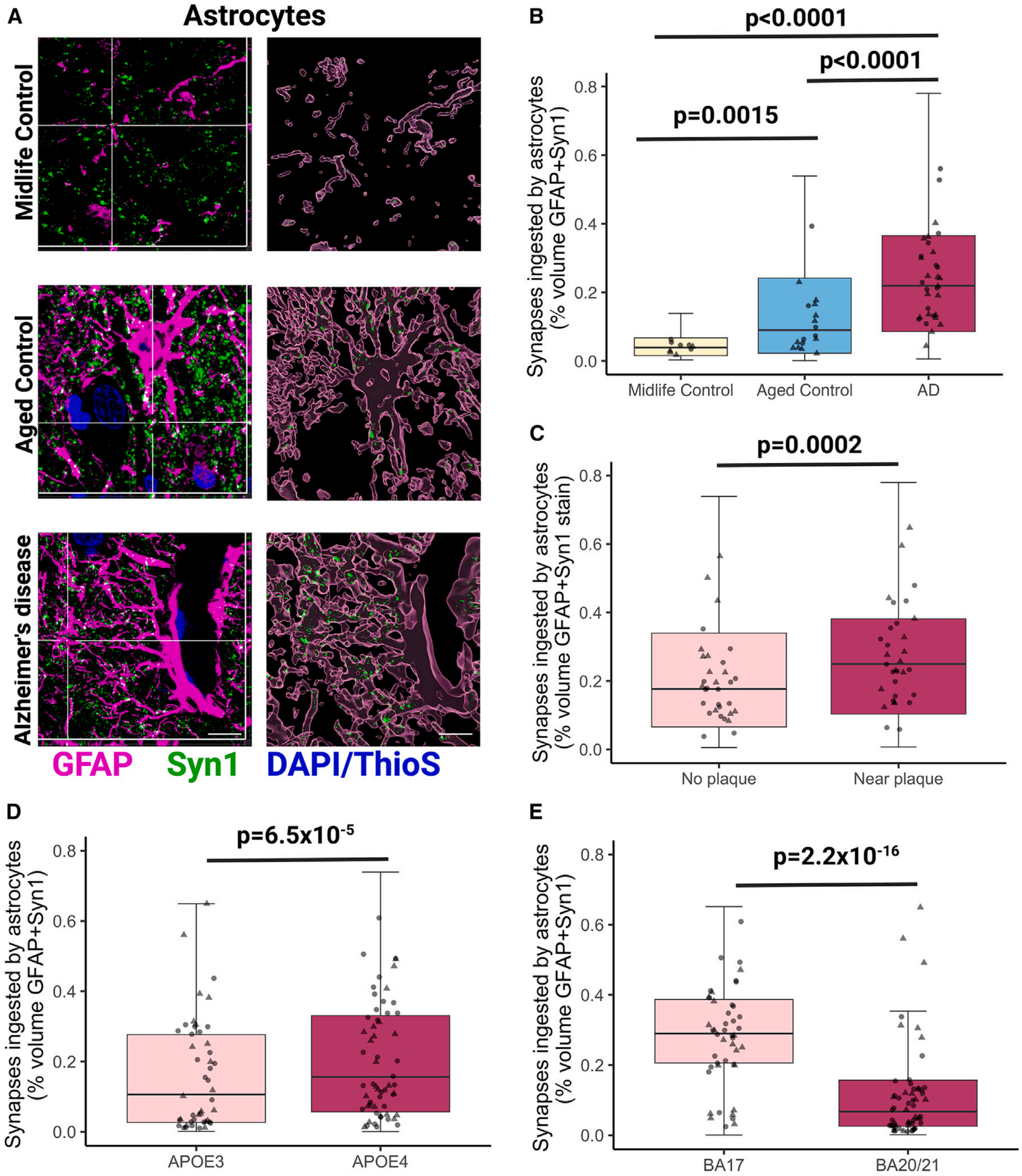

Figure 1. Astrocytes ingest more synapses in Alzheimer's disease compared with midlife and aged controls

(A) Confocal images of immunostaining with orthogonal views (left) and three-dimensional reconstructions of stacks (right) of midlife control, aged control, and Alzheimer's disease showing ingestion of synapsin 1 (Syn1, green) by astrocytes (GFAP, magenta) in human brain sections. Aβ plaques and nuclei are counterstained with Thioflavin S and DAPI, respectively, shown in blue. Only ingested Syn1 is shown in 3D Imaris reconstructions. Representative images are from BA17. Scale bars represent 5 μm.

(B) Quantification of synapsin 1 ingested by GFAP-positive astrocytes showed significantly increased levels in AD compared with both midlife controls (post hoc Tukey corrected tests after linear mixed effects model $t = 8.138$, $p < 0.0001$) and aged controls ($t = 4.997$, $p < 0.0001$), and also between midlife controls to aged controls ($t = 3.68$, $p = 0.0015$).

(C) Statistically significant increase of synapsin 1 ingestion by astrocytes near Aβ plaques in AD cases ($F[1,1202.02] = 13.6$, $p = 0.0002366$).

(D) The *APOE4* genotype was associated with an increase in synapsin 1 colocalization inside GFAP-positive astrocytes ($F[1,78.36] = 17.81$, $p = 6.5 \times 10^{-5}$).

(legend continued on next page)

RESULTS

We examined human postmortem tissue from individuals with late-stage AD (n = 32), age-matched control cases (n = 18), and midlife control cases (n = 10) without neurological disorders in two brain areas, the inferior temporal lobe (temporal cortex, BA20/21), which contains substantial Aβ and tau pathology, and the primary visual cortex (occipital cortex, BA17), which is affected later in dementia and contains less pathology than temporal cortex even at end stages of disease (information for human cases found in Table S1).

To determine whether astrocytes ingest synapses in human brain, we examined colocalization between GFAP and Syn1 (Figure 1A) and observe a 2.1-fold increase in AD compared with age-matched control brain (Figure 1B), indicating astrocytes ingest more synapses in AD than control ($p < 0.0001$). Further, there is a 2.7-fold increase in the volume occupied by synapses and GFAP between midlife and healthy aging ($p = 0.0015$), indicating that astrocyte ingestion of synapses increases during age and further increases in AD ($p < 0.0001$) (Figure 1B). Astrocytes ingest more synapses in the proximity of Aβ plaques in BA20/21 ($p = 0.0004$) but not in BA17 in AD brain (Figures 1C and S1). Also, we observe significantly higher astrocytic ingestion of Syn1 in individuals who carry the *APOE4* allele than those who do not ($p = 6.5 \times 10^{-5}$) (Figures 1D, S1C). Moreover, there is more colocalization of astrocytes and synapses in BA17 than BA20/21 ($p < 0.0001$) (Figure 1E), which occurs both in aged control and in AD cases (Figure S1C). When ingestion was normalized to GFAP burden, there was still a significant effect of cohort ($p < 0.0001$) with both AD and aged controls having more synapse colocalization with astrocytes than midlife controls in post hoc tests (Figure S1D). Interestingly, women had significantly more synaptic ingestion by astrocytes when normalized to GFAP burden ($p = 0.015$). To confirm synaptic ingestion by astrocytes with a sub-diffraction limit technique, we re-analyzed a recently published array tomography dataset,[24] and confirmed at sub-synaptic resolution that synapses are within GFAP-positive astrocytes (Figures S1J and S1K).

We also assessed internalization of synapses by microglia using Syn1 colocalization with the microglial lysosomal marker CD68 (Figure 2A), which was also confirmed by super-resolution Airyscan microscopy (Figure S1E). CD68 labels both microglia and macrophages, and in the cortical neuropil regions examined from our cases, we observed that CD68-positive cells were also positive for IBA1 and the microglial marker TMEM119[24,25] (Figure S1G). We found a significant increase in the volume of colocalization between CD68 and Syn1 in AD compared with aged controls ($p = 0.0024$) and in AD compared with midlife controls ($p = 0.0096$), suggesting increased synaptic ingestion by microglia in disease (Figure 2B). The increased synaptic ingestion by microglia in AD was present in both BA17 and BA20/21 (Figure S1H). Unlike astrocyte ingestion, there was no increase in microglial ingestion of synapses between midlife and healthy aging ($p = 0.625$) (Figure 2B). Moreover, in AD brain samples, this ingestion was higher in the presence of Aβ plaques ($p = 8.15 \times 10^{-7}$) (Figure 2C) and in individuals with the *APOE4* genotype ($p = 0.02$) (Figures 2D and S1H). Similar to astrocytes, there is more colocalization of microglia and synapses in BA17 than BA20/21 ($p < 0.0001$) (Figures 2E and S1H). As expected, we observed microgliosis in AD brain with a 1.9-fold increase in CD68 volume across both brain areas. When synaptic colocalization with CD68 was normalized to CD68 volume, no differences were seen between AD and aged control brain, indicating that the increased colocalization (reflective of ingestion) is at least partly driven by microgliosis and/or microglial hypertrophy (Figure S1I). These data show that microglia contain synaptic protein in human brain with more ingestion in AD, more near plaques, and more in *APOE4* carriers.

To further validate microglial ingestion of synapses, we immunostained for the microglial-specific marker P2Y12, which confirmed synaptic colocalization with this marker (Figure 3A). Since synapsin 1 can be observed in axons as well as synapses, we confirmed synaptic ingestion by both astrocytes and microglia using synaptophysin, a more specific presynaptic vesicle marker (Figure 3A). We also observed excitatory postsynaptic densities (PSD95) and inhibitory presynaptic proteins GAD65/67 within astrocytes and microglia (Figures S2A and S2B). The synaptic ingestion we observed in postmortem samples could be due to specific phagocytosis of synapses from living neurons or more general clearance of dead or dying neurons by glia. To investigate this possibility, we co-stained synaptic protein synaptophysin, neuronal neurite protein MAP2, astrocytes, and microglia (Figure 3A). Similar to our previous experiments, we observed significantly higher colocalization of synaptophysin with both astrocytes ($p = 0.001$, Figure 3B) and microglia ($p = 0.047$, Figure 3C). In AD cases, triple colocalization of MAP2 with synaptophysin and astrocytes showed a 10-fold lower colocalization than synaptophysin with astrocytes (Figure 3D), and similarly MAP2 with synaptophysin and microglia showed a 44-fold lower colocalization compared with synaptophysin with microglia (Figure 3E), suggesting synaptic ingestion occurs predominantly in the absence of neurite ingestion. We also did not observe axonal neurofilament ingestion by astrocytes and microglia (Figure S2C).

In terms of relevance of glial ingestion of synapses to synapse loss, we previously characterized a subset of these same human brain donors with array tomography to accurately measure synapse density (n = 4 midlife, 10 aged control, AD 16, data from King et al.[4] and Colom-Cadena et al.[5]). In this subset of cases, the synapse density negatively correlated with astrocyte ingestion of synapses in BA20/21 (Figure S1B, correlation coefficient −0.68, $p < 0.0001$). Microglial engulfment of synapses did not correlate with synapse density in either brain region (Figure S1F).

(E) BA17 (primary visual cortex) was associated with higher levels of synapsin 1 colocalization inside GFAP-positive astrocytes compared with BA20/21 (inferior temporal cortex) ($F[1,2228.59] = 1382.4$, $p = 2.2 \times 10^{-16}$). Statistical comparisons were made using ANOVA after linear mixed effects model on Tukey transformed data with case as a random effect and disease, brain region, *APOE4* status, gender, and age as fixed effects. Untransformed data are presented in graphs (B)–(E). All data are included in boxplots and case medians are shown in points. Data in this figure are pooled from both BA17 and BA20/21. Males are represented by circles and females by triangles. Biological replicates were human brain donors: n = 10 midlife controls, 19 aged controls, AD 31 cases.

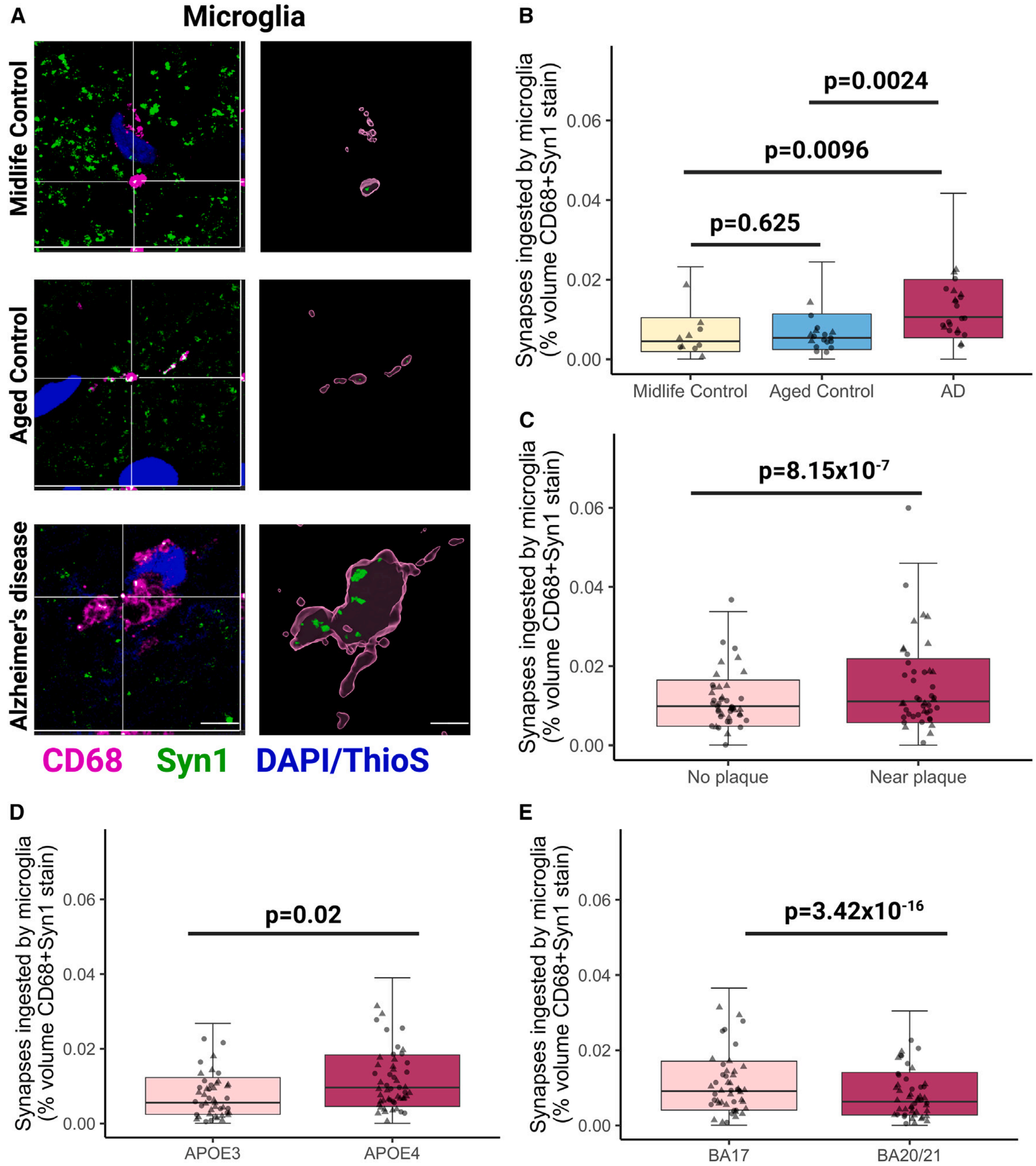

Figure 2. Microglia ingest more synapses in Alzheimer's disease compared with midlife and aged controls

(A) Confocal images of immunostaining with orthogonal views (left) and three-dimensional reconstructions of stacks (right) of midlife control, aged control, and Alzheimer's disease showing ingestion of synapsin 1 (Syn1, green) by microglia (CD68, magenta) in human brain sections. Aβ plaques and nuclei are counterstained with Thioflavin S and DAPI, respectively, shown in blue. Only ingested Syn1 is shown in 3D Imaris reconstructions. Representative images are from BA17. Scale bars represent 5 μm.

(B) Quantification of synapsin 1 ingested by CD68-positive microglia showed significantly increased levels in AD compared with midlife (Tukey corrected post hoc test $t = 3.56$, $p = 0.0024$) and aged controls ($t = 3.09$, $p = 0.0096$). No statistical difference was seen between midlife controls and aged control ($t = 0.93$, $p = 0.625$). Data here are pooled from both BA17 and BA20/21, sample size corrected by mixed effected linear model.

(C) Statistically significant increase of synapsin 1 ingestion by microglia near Aβ plaques in AD cases (ANOVA $F[1,881.57] = 20.74$, $p = 6.01 \times 10^{-6}$).

(D) The *APOE4* genotype was associated with an increase in synapsin 1 colocalization inside CD68-positive microglia ($F[1,42.86] = 5.84$, $p = 0.02$).

(legend continued on next page)

Having shown in postmortem human tissue that there is more synaptic protein located within microglia and astrocytes in AD compared with control brains, we next sought to determine whether synapses derived from human AD brains are more readily internalized by glia in dynamic *ex vivo* assays. We used snap-frozen samples from the temporal lobe (Brodmann area 38, or BA38) of control and AD cases to prepare synaptoneurosomes and synaptosomes in synapse-enriched fractions (SEFs), as previously validated by our lab[26] and others.[27] Western blotting showed the synaptoneurosomes and synaptosomes had significantly higher levels of both synaptophysin (Sy38) and postsynaptic density 95 (PSD-95) in the SEFs compared with total brain homogenate, as well as a significant de-enrichment of the nuclear marker histone H3 (Figures S3A–S3C, S3E, and S3F). Synaptic integrity was also validated by electron microscopy (Figure S3G). We observed lower levels of synaptophysin in AD brain homogenates and synaptoneurosomes compared with brains from age-matched controls without dementia, reflecting the known synapse loss that occurs in AD (Figure S3D).

Synaptoneurosomes and synaptosomes were conjugated to pHrodo-Red succinidyl ester, enabling visualization of synapses internalized within the acidic phago-lysosomal compartment during phagocytosis.[28,29] We challenged cultured GFAP-expressing human and mouse astrocytes (Figures S4A and S4B) with pHrodo-Red labeled synaptoneurosomes from AD or control brain to confirm they phagocytose synapses (Figures 4A and 4B, Videos S1 and S2). We observed that cultured human astrocytes phagocytose synaptoneurosomes from AD brain both more ($p = 8.47 \times 10^{-4}$) and faster ($p = 8.25 \times 10^{-14}$) than those from controls (Figures 4C and 4D). Similarly, AD synaptosomes were also ingested more than control synaptosomes by human astrocytes (Figure S3H). Primary mouse astrocytes similarly ingested AD synapses both more ($p = 8 \times 10^{-4}$) and faster ($p = 2 \times 10^{-16}$) than controls (Figures 4E and 4F). Importantly, phagocytosis of synaptoneurosomes was completely suppressed in astrocytes treated with cytochalasin D (CytD), a potent inhibitor of actin polymerization that prevents phagocytosis (Figures 4C, 4E, S4C, and S4D).

We next tested whether microglia also ingest AD synapses differently from controls. The BV2 murine microglial cell line was used to validate phagocytosis of pHrodo-tagged synaptoneurosomes where clear uptake of synaptoneurosomes into acidic subcellular compartments was observed, while this was diminished by CytD (Figures S4E and S4F). We then tested uptake of synaptoneurosomes from control or AD brains by primary human microglia isolated from peritumoral tissue extracted during glioblastoma surgeries (n = 11 patient donors; Table S2). Microglia were isolated by immunomagnetic separation, as previously validated for mice by our group,[30] and human cells were shown to express the microglia-specific marker TMEM119 (Figure 5A). Human microglia in culture were exposed to AD or control synaptoneurosomes labeled with pHrodo and imaged using live microscopy (Figures 5B and 5C, Videos S3 and S4). A significantly greater proportion of human microglia phagocytosed AD synaptoneurosomes compared with controls ($p = 0.027$) and this process also occurred faster ($p = 2 \times 10^{-16}$) (Figures 5D and 5E). There were no effects of gender, age, or resection brain region for the microglial surgical donors. We also observed similar results from adult primary microglia from non-diseased temporal cortex of a neurosurgical biopsy due to epilepsy (Figures S5A–S5C), as well as from human pluripotent stem-cell-derived microglia-like cells (Figures S5D–S5H). Given the inter-person variability of human microglia, we also challenged adult primary mouse microglia with the human synaptoneurosomes. Similar to the human microglia, murine microglia also ingested AD synaptoneurosomes both more ($p = 0.01$) and faster ($p < 0.0001$) than control synaptoneurosomes (Figures 5F and 5G, effect of disease of synapse donor: $F[1,2.88E21] = 102.39$, $p < 0.0001$; effect of incubation time $F[18,inf] = 98.10$, $p < 0.0001$). Primary mouse microglia also preferentially ingested AD synaptosomes compared with control synaptosomes (Figure S3I). Interestingly, cultured astrocytes phagocytosed synapses more slowly than microglia, with maximum phagocytosis occurring at around 60 min in microglia and around 48 h in astrocytes. Last, we excluded the possibility that AD-derived synapses interfere with the phago-lysosomal compartment to account for this increase in ingestion by running degradation assays in cultured astrocytes and microglia showing effective degradation of both AD and control synaptoneurosomes (Figures S6A–S6C).

One possible mechanism for tagging synapses for ingestion is via MFG-E8, a phosphatidylserine-recognizing opsonin. Astrocytes, microglia, and macrophages produce MFG-E8 both *in vitro* and *in vivo,* which can bind to exposed phosphatidylserine on living neurons challenged with either Aβ or p-tau, facilitating phagocytosis via binding integrins on phagocytes.[31–35] We have previously observed increased levels of MFG-E8 at the synapse in AD individuals compared with controls by unbiased proteomic screening.[36] Here, we have validated that the AD synaptoneurosomes tested in the phagocytosis assays also express higher levels of MFG-E8 compared with control samples ($p = 0.01$) by quantitative dot blots (Figures 6A–6C). Thus, we tested the hypothesis that blocking the integrin binding site on MFG-E8 would modulate phagocytosis of AD and control synaptoneurosomes. Synaptoneurosomes from AD and control brain were pre-incubated with an antibody to block the integrin binding domain of MFG-E8 (or immunoglobulin [Ig]G1 isotype control) before applying them to cultured human microglia or astrocytes. In human astrocyte cultures (Figure 6D), anti-MFG-E8 antibody treatment significantly reduced phagocytosis of AD synaptoneurosomes ($p = 0.0011$) compared with both untreated and IgG1 (isotype control for anti-MFG-E8 antibody) incubation ($p = 0.0384$) (Figure 6E and S5I). When astrocytes were exposed to control synapses, there was no effect of either MFG-E8 or IgG1 treatment on phagocytosis. This provides a mechanism

(E) BA17 (primary visual cortex) was associated with higher levels of synapsin 1 colocalization inside CD68-positive microglia compared with BA20/21 (inferior temporal cortex) ($F[1,1973.76] = 67.82$, $p = 3.42 \times 10^{-16}$). Statistical comparisons were made using post hoc Tukey test or ANOVA after linear mixed effects model on Tukey transformed data with case as a random effect and disease, brain region, *APOE4* status, gender, and age as fixed effects. Untransformed data are presented in graphs (B)–(E). Males are represented by circles and females by triangles. Biological replicates were human brain donors: n = 10 midlife controls, 17 aged controls, AD 22 cases.

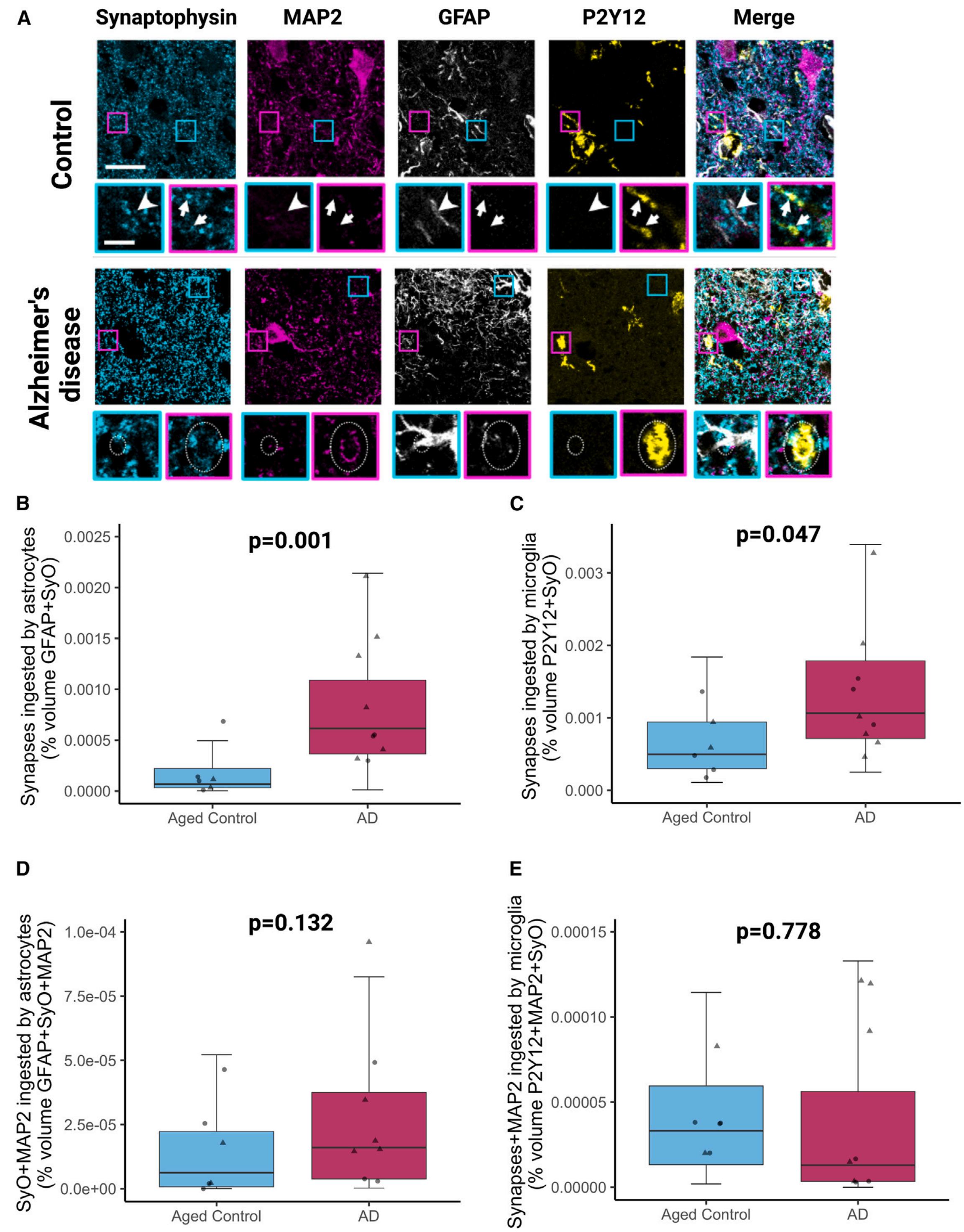

Figure 3. Astrocytes and microglia ingestion of synapses in the absence of neuronal neurite ingestion

(A) Staining presynaptic terminals with synaptophysin, neuronal neurites with MAP2, astrocytes with GFAP, and microglia with P2Y12 reveals synaptic ingestion by astrocytes (cyan boxes, arrowheads) and microglia (magenta boxes, arrows) in the absence of MAP2 staining in control and AD brain. In AD, MAP2 positive neuronal protein can also be observed in astrocytes (cyan boxes, dotted ovals) and microglia (magenta boxes, dotted ovals). Large panels are maximum intensity projections of confocal image stacks. Insets are single sections to demonstrate colocalization. Scale bars represent 20 μm in large panels, 5 μm in insets.

(B) Quantification of synaptophysin (SyO) ingested by GFAP-positive astrocytes showed significantly increased levels in AD compared with aged controls (-ANOVA after linear mixed effects model with cohort, gender, and age as fixed effects and case as a random effect $F[1,13.02] = 17.38$, $p = 0.001$).

(legend continued on next page)

by which astrocytes ingest AD-specific synapses and a pathway that can be targeted to modulate phagocytosis. In human brains, astrocytes abundantly express the integrin receptor α5β5, which is known to bind MFGE8.[37] Therefore, we used an antibody to block this receptor and test its effects in ingestion of synaptoneurosomes. We found that anti-α5β5 treatment on human astrocytes resulted in significantly reduced phagocytosis of AD ($p = 0.006$), but not control ($p = 0.0758$) synaptoneurosomes compared with untreated cells (Figure 6F). This suggests that both MFG-E8 and its integrin receptor α5β5 are important molecular pathways in astrocytic ingestion of synapses in AD.

We also challenged human cultured microglia with synaptoneurosomes treated with the anti-MFG-E8 antibody or IgG1 isotype control (Figure 6G) as above. MFG-E8 antibody treatment reduced phagocytosis of AD synaptoneurosomes compared with untreated ($p < 0.0001$) and IgG1 isotype control ($p = 0.0208$). We also found that AD synaptoneurosome phagocytosis was decreased by IgG isotype alone ($p < 0.0001$) compared with non-antibody-containing vehicle, despite prior blocking of low affinity Fc receptors (with anti-CD16/32) on microglia in all conditions (Figure 6H). Anti-MFG-E8 treatment attenuated microglial phagocytosis of AD synapses significantly more than IgG1 treatment ($p = 0.0208$), suggesting MFG-E8 acts as a more specific opsonin on synapses than IgG1. Anti-MFG-E8 treatment did not affect microglial phagocytosis of control brain-derived synaptoneurosomes, similar to astrocytes (Figure 6H). The absence of an IgG1 effect in astrocyte cultures above is consistent with their lacking expression of Fc receptors. Overall, blocking synaptic MFG-E8 on AD synapses also reduced phagocytosis by human microglia.

DISCUSSION

In this study, we have demonstrated that microglia and astrocytes in human AD brains contain more synaptic protein than in control brains, and that *in vitro* these cells preferentially ingest AD-derived synapses compared with controls. This builds upon previous evidence that microglia and astrocytes are capable of containing synaptic material in AD,[38–40] and further indicates that the synapse loss we have observed in the same cohort of brain donors[4,5] may be influenced by glial ingestion. We speculate that these differences in synaptic ingestion are due to AD-specific mechanisms, as in schizophrenia, a disease also characterized by reduced synaptic levels where microglia partake in increased synaptic phagocytosis.[41] We have found that there are no differences in synaptic ingestion by microglia in postmortem tissue of people with and without schizophrenia.[42] In AD, this could in theory be beneficial in clearing dysfunctional or dead synapses or could be harmful in contributing to synaptic degeneration and removal of functional synapses. It is possible that both are true with a differing balance of beneficial and harmful contributions of glia to synaptic phenotypes at different stages of disease and in different parts of the brain. Indeed, there is evidence that when microglia phagocytose amyloid beta due to manipulation of TDP-43, synapses are also phagocytosed,[43] which demonstrates that a potentially beneficial action of microglia, namely clearance of pathological proteins, may be linked to the potentially detrimental effect of synapse removal.

Here, we have shown that MFG-E8 may contribute to the glial-mediated synapse ingestion observed in AD. MFG-E8 protein is likely increased on AD synapses due to binding phosphatidylserine, which is a well-known microglial opsonin that has been observed to play a role in neuron phagocytosis by microglia in mouse cultures.[31] Phosphatidylserine performs many signaling roles and is a major component of all neuronal plasma membranes. *MFGE8* is expressed by astrocytes and microglia in cortical gray matter,[37,44,45] while *ITGAV* and *ITGBV* are also expressed in these cell types, which is important, as MFG-E8 is known to bind glial integrin α5β5 encoded by these genes.[46] Notably in human brain, *ITGB3* was expressed only at very low levels in astrocytes and microglia, indicating that the MFG-E8 effect we observe in human astrocytes is likely driven by interaction with integrin α5β5 not α5β3, which it can also bind.[37] Published single nucleus sequencing data from AD vs. control brain confirm that *MFGE8* is expressed in astrocytes and is significantly upregulated in AD vs. control astrocytes.[45] This suggests that AD brain-derived synapses already partially tagged with MFG-E8 as we showed previously[36] could be further opsonized by astrocytes secreting MFG-E8 during culture, thus reinforcing this pathway as a mechanism for astrocyte-mediated synapse uptake. *ITGB5* is expressed in microglia and astrocytes in these data and in AD it is upregulated in astrocytes but not microglia. Interestingly, this dataset also showed that *ITGAV* is expressed in both microglia and astrocytes and is downregulated in AD in both cell types.[45] *MFGE8*, *ITGB5*, and *ITGAV* mRNA are all found in nuclei as well as cell somata, making the data from single nuclear sequencing studies of human postmortem brain likely a reliable reflection of the expression levels of these genes.[47]

With a clearer understanding of glia-synapse interactions, there is potential to develop effective therapeutics to protect synapse function. Drugs that attenuate astrocytic and microglial phagocytic activity and anti-complement therapies have shown some potential to reduce AD-associated pathology in mice, notably limiting synapse loss and rescuing cognitive deficits.[16,22,48] This is very important, as it suggests glial cells do not only clear dystrophic or degenerating neurons,[39,40] but may also eliminate healthy ones. Our data here show the relevance of this process in human AD brain and glial cells, and reveal a potential mechanism by which augmented AD synapse uptake can occur.

(C) Quantification of SyO ingested by P2Y12-positive microglia showed significantly increased levels in AD compared with aged controls (ANOVA after linear mixed effects model $F[1,13.007] = 4.8$, $p = 0.047$).
(D) Quantification of SyO and MAP2 ingested by GFAP-positive astrocytes showed no significant differences between AD and aged controls (ANOVA after linear mixed effects model $F[1,13.01] = 2.58$, $p = 0.1324$).
(E) Quantification of SyO and MAP2 ingested by P2Y12-positive microglia showed no significant differences between AD and aged controls (post hoc Tukey corrected tests after linear mixed effects model $F[1,12.98] = 0.08$, $p = 0.778$). Biological replicates were human brain donors: n = 5 aged controls, AD 8 cases. Males are represented by circles and females by triangles.

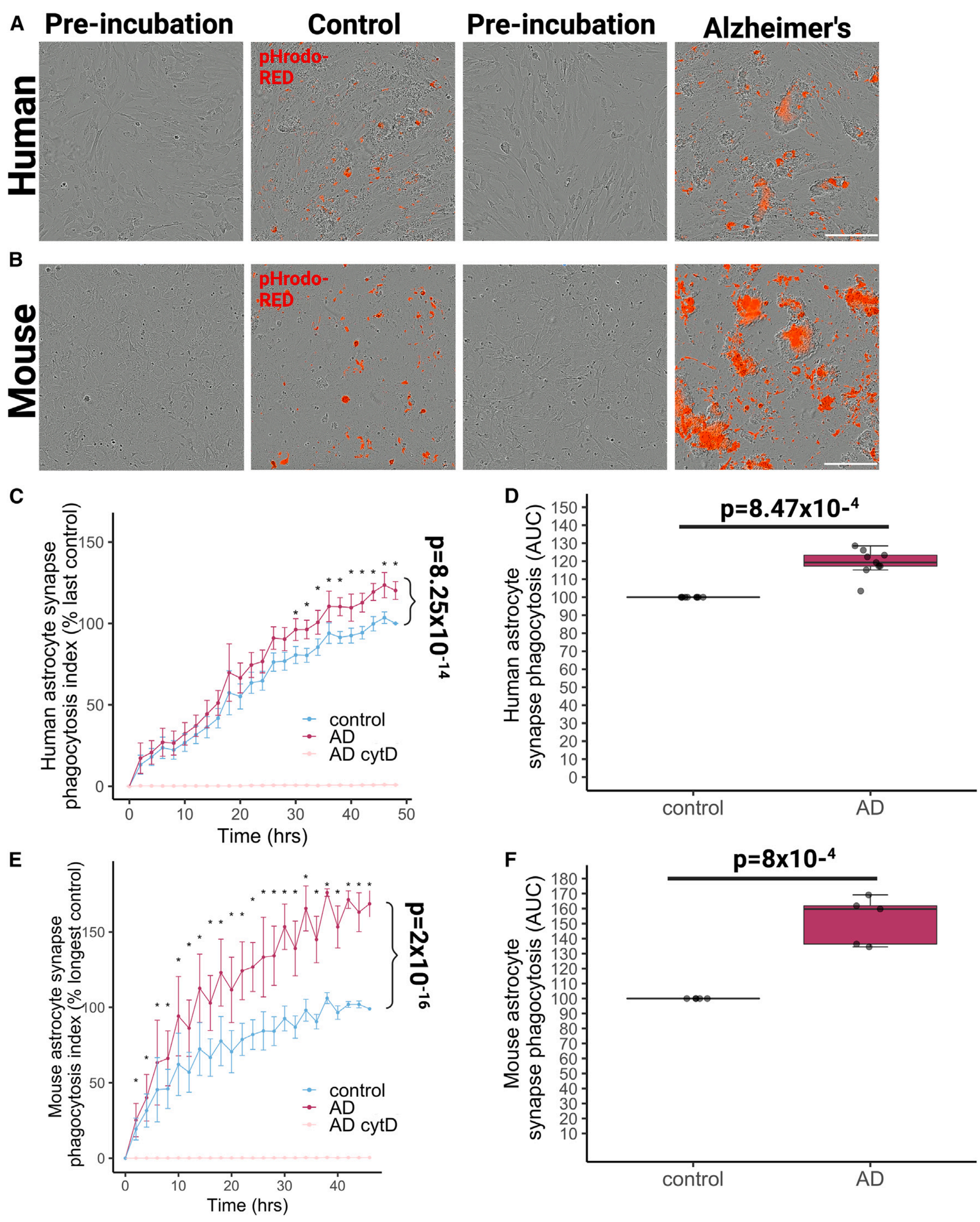

Figure 4. Human and mouse astrocytes ingest human AD synapses more than control synapses in culture

(A) Live-imaging assay of primary human fetal astrocytes shows astrocytes phagocytose pHrodo-Red-labeled human synaptoneurosomes from control and Alzheimer's disease (AD) brains after treatment (48 h). Scale bar represents 200 μm.

(B) Live-imaging assay of primary mouse embryonic astrocytes shows astrocytes phagocytose pHrodo-Red-labeled human synaptoneurosomes from control and AD brains after treatment (48 h). Scale bar represents 200 μm.

(C) The phagocytosis index curves normalized to within experiment control at the last time point show that human astrocytes (n = 8 independent culture replicates) phagocytose AD synapses more and faster than controls (ANOVA after linear mixed effects model with disease status of synapse donor and incubation time as fixed effects and experimental replicate as random effect, effect of disease status $F[1,370.03] = 50.25$, $p < 0.0001$, effect of time $F[24,370.09] = 85.45$, $p < 0.0001$). Asterisks represent significant post hoc Tukey corrected tests between AD and control at the time points indicated.

(legend continued on next page)

Limitations of the study

The first three figures of this study are limited by the use of human postmortem tissue, which provides a snapshot at the end stage of AD and does not allow longitudinal study of glial-mediated ingestion of synapses nor deeper mechanistic investigations of these mechanisms by pharmacological or genetic interventions. As such, all human mechanistic data here were collected *in vitro,* which is not ideal for investigating glial cells, as these cells are sensitive to homeostatic changes. Furthermore, human microglia were collected from peritumoral areas or regions near an epileptic focus during brain surgery, which could also affect their function. Due to the processing of donated brains, it is difficult to study synaptic ingestion by glial cells in humans using super-resolution microscopy techniques. Last, unlike mice, there are many independent variables in human studies, including genetics, environmental factors, medications, and cause of death, which can all influence brain function. Despite these limitations, our work provides evidence from human brain and living human brain cells that astrocytes and microglia ingest synapses and hint at a mechanism that may be a future target to modulate this ingestion.

STAR★METHODS

Detailed methods are provided in the online version of this paper and include the following:

- KEY RESOURCES TABLE
- RESOURCE AVAILABILITY
  - Lead contact
  - Materials availability
  - Data and code availability
- EXPERIMENTAL MODEL AND SUBJECT DETAILS
  - Animals
  - Human postmortem tissue
  - BV2 microglia phagocytosis assay
  - Primary mouse microglia
  - Primary human (GBM) microglia isolation
  - Primary human (epilepsy case) microglia isolation
  - Human pluripotent stem cell derived microglia cultures
  - Primary mouse astrocyte isolation
  - Primary human astrocyte line
- METHOD DETAILS
  - Immunohistochemistry for paraffin embedded human tissue
  - Confocal microscopy and image analysis
  - Airyscan confocal microscopy
  - Synaptoneurosome preparation
  - Synaptosome preparation
  - Electron microscopy
  - Protein extraction
  - Micro BCA
  - Western blot
  - Dot blots
  - Synaptoneurosome and synaptosome labeling with pHrodo Red-SE
  - IncuCyte phagocytosis assay on astrocytes
  - MFG-E8 and integrin (α5β5) antibody blocking *in vitro*
  - Degradation assay
  - Immunofluorescence of cultured cells
- QUANTIFICATION AND STATISTICAL ANALYSIS

SUPPLEMENTAL INFORMATION

Supplemental information can be found online at https://doi.org/10.1016/j.xcrm.2023.101175.

ACKNOWLEDGMENTS

We wish to thank our human tissue donors and their families for making this work possible. We gratefully acknowledge Edinburgh Neuroscience and the FENS-Kavli Network of Excellence for facilitating collaborations leading to this work. We also wish to thank the MRC Edinburgh Brain Bank for providing human tissue and the group of Prof. Nancy Ip, Prof. Oscar Harari, and Prof. Mark Fiers for sharing their single nucleus sequencing data freely with their publications. We would like to thank the Lothian Bioresource for facilitating biopsy tissue collection. We would like to thank Dr. Jason Early and Prof. David Lyons at CDBS and MS Society Edinburgh for assisting with Airyscan microscopy. We would like to thank Dr. Anisha Kubasik-Thayil at the IMPACT Imaging Facility for use of Imaris at the University of Edinburgh. We would also like to acknowledge acquisition of electron microscopy images at the Roslin Institute 3D Electron Microscopy Facility. Figures were created using Biorender. This work was funded by the UK Dementia Research Institute, which receives its funding from DRI Ltd, funded by the UK Medical Research Council, Alzheimer's Society, and Alzheimer's Research UK, the European Research Council (ERC) under the European Union's Horizon 2020 research and innovation program (grant agreement no. 681181), Alzheimer's Research UK, and the Scottish Government Chief Scientist Office (ARUK SPG2013-1), a Wellcome Trust-University of Edinburgh Institutional Strategic Support Fund, MND Scotland, and Alzheimer's Society. Electron micrographs were obtained at the Roslin Institute 3D Electron Microscopy Facility (funded by BBSRC Institute Strategic Program support).

AUTHOR CONTRIBUTIONS

Conceptualization: M.T., M.J.D.D., C.M.H., B.W.M., and T.L.S.-J. Methodology: M.T., M.J.D.D., C.D., D.K., P.B., S.M., B.V., K.P., A.J.S., J.B., E.D., J.B., R.K.H., J.T., C.L., J.K., D.S., C.S., V.E.M., R.T.K., G.E.H., C.M.H., P.M.B., B.W.M., and T.L.S.P. Investigation: M.T., M.J.D.D., C.D., B.V., K.P., M.H., E.D., J.B., J.M., M.H., and T.L.S.J. Visualization: M.T. and T.L.S.J. Funding acquisition: M.T., B.W.M., and T.L.S.-J. Project administration: B.W.M. and T.L.S.-J. Supervision: M.T., M.J.D.D., C.M.H., B.W.M., and T.L.S.-J. Writing – original draft: M.T. and T.L.S.-J. Writing – review & editing: All authors.

(D) Examining the area under the curve confirms that AD synapses are phagocytosed more by human astrocytes than control synapses (ANOVA after linear mixed effects model with disease status of synapse donor as fixed effect and experimental replicate as random effect $F[1,9.60] = 61.14$, $p = 1.83 \times 10^{-5}$).
(E) Mouse astrocytes (E and F, n = 5 independent culture replicates) also ingest AD synapses more and faster than controls (effect of disease of synapse donor $F[1,163.06] = 230.98$, $p < 0.0001$; effect of incubation time $F[25,163.17] = 28.40$, $p < 0.0001$). Asterisks represent significant post hoc Tukey corrected tests between AD and control at the time points indicated.
(F) Examining the area under the curve confirms that AD synapses are phagocytosed more by mouse astrocytes than control synapses ($F[1,4.80] = 54.65$, $p = 0.0008$). CytD, which inhibits phagocytosis, completely prevented synapse ingestion.

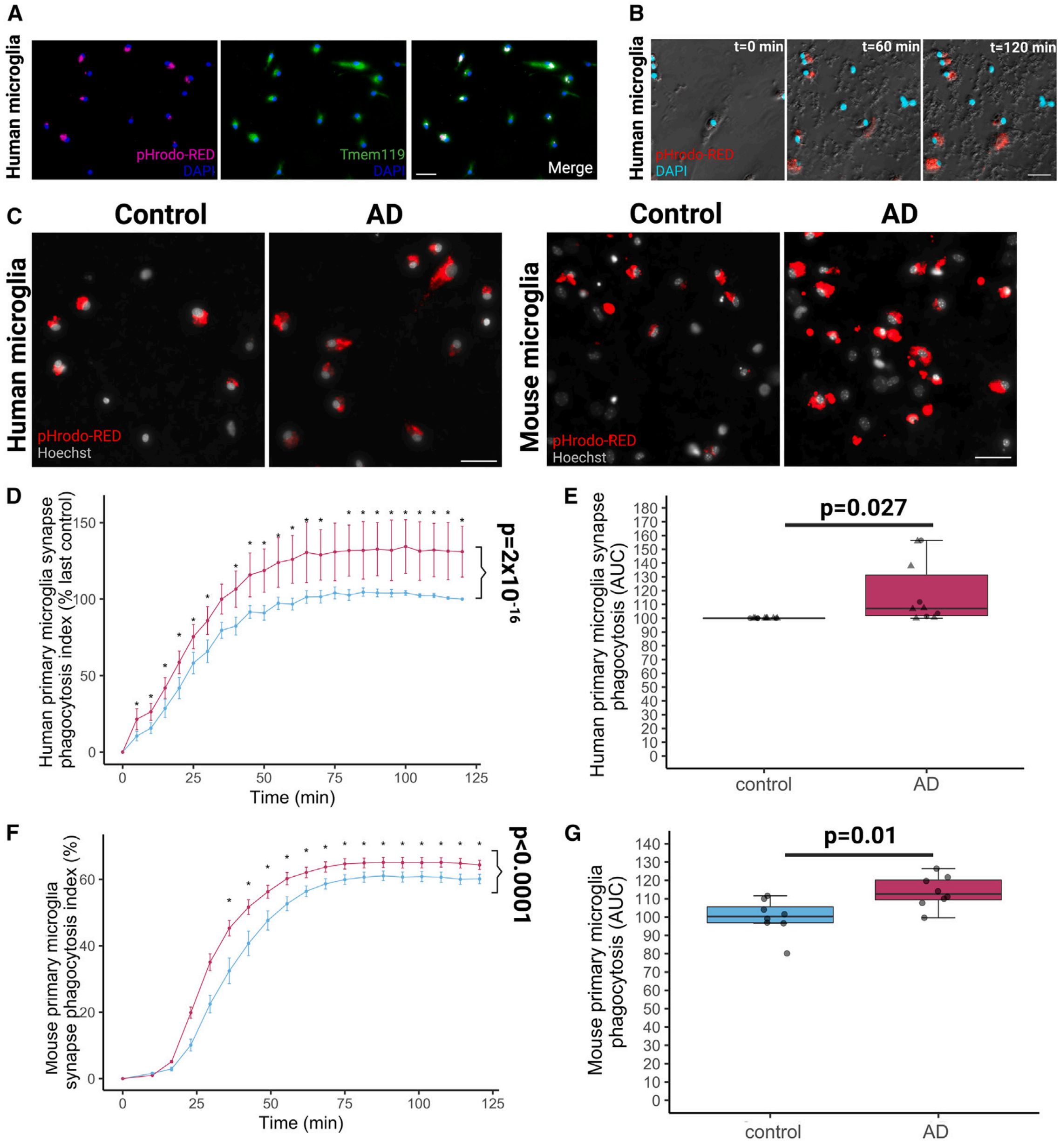

Figure 5. Human and mouse microglia ingest human AD synapses more than control synapses in culture

(A) Immunocytochemistry on fixed microglia grown from human peritumoral tissue resected during neurosurgery shows they express the microglial-specific marker TMEM119 and engulfed pHrodo-Red-labeled human synaptoneurosomes when these were applied 2 h before fixation. Scale bar represents 20 μm.

(B) Live imaging of human microglia (phase) with DAPI (cyan) and pHrodo-Red confirms live phagocytosis of human synapses over 2 h. Scale bar represents 20 μm.

(C) Live imaging of human microglia with nuclear marker Hoechst and pHrodo-Red confirms that these primary cultured microglia engulf synapses derived from human AD and control brain. Scale bar represents 20 μm.

(D) Quantification of experiments from n = 11 neurosurgical donors (each a biological replicate) shows that the phagocytosis index normalized to the final value for the control condition for each experiment increases over time and that AD synapses are phagocytosed more and faster than control synapses (ANOVA after linear mixed effects model on square root transformed data with synaptoneurosome donor disease status, incubation time, neurosurgical brain region, microglial donor gender, and microglial donor age as fixed effects and donor as a random effect, effect of disease status of synapse donor $F[1,396.09] = 122.74$, $p < 2 \times 10^{-16}$, effect of incubation time $F[24,396.25] = 119.49$, $p < 2 \times 10^{-16}$).

(E) Quantifying the area under the curve of the phagocytosis index confirms more phagocytosis of AD than control synapses (*ANOVA after linear mixed effects model with synaptoneurosome donor disease status, incubation time, neurosurgical brain region, microglial donor gender, and microglial donor age as fixed effects and donor as a random effect, effect of disease status of synapse donor $F[1,12] = 6.38$, $p = 0.027$).

(legend continued on next page)

DECLARATION OF INTERESTS

T.L.S.-J. is on the Scientific Advisory Board of Cognition Therapeutics and Scottish Brain Sciences and receives collaborative grant funding from two industry partners. None of these had any influence over the current paper.

INCLUSION AND DIVERSITY

One or more of the authors of this paper self-identifies as a member of the LGBTQ+ community. We support inclusive, diverse, and equitable conduct of research.

Received: August 19, 2022
Revised: January 30, 2023
Accepted: August 7, 2023
Published: August 30, 2023

REFERENCES

1. Braak, H., and Braak, E. (1995). Staging of Alzheimer's disease-related neurofibrillary changes. Neurobiol. Aging *16*, 271–278, discussion 278-284. https://doi.org/10.1016/0197-4580(95)00021-6.
2. Thal, D.R., Rüb, U., Orantes, M., and Braak, H. (2002). Phases of A beta-deposition in the human brain and its relevance for the development of AD. Neurology *58*, 1791–1800. https://doi.org/10.1212/wnl.58.12.1791.
3. Pickett, E.K., Herrmann, A.G., McQueen, J., Abt, K., Dando, O., Tulloch, J., Jain, P., Dunnett, S., Sohrabi, S., Fjeldstad, M.P., et al. (2019). Amyloid beta and tau cooperate to cause reversible behavioral and transcriptional deficits in a model of Alzheimer's disease. Cell Rep. *29*, 3592–3604.e5. https://doi.org/10.1016/j.celrep.2019.11.044.
4. King, D., Holt, K., Toombs, J., He, X., Dando, O., Okely, J.A., Rose, J., Gunn, C., Correia, A., Montero, C., et al. (2022). Synaptic resilience is associated with maintained cognition during ageing. Alzheimers Dement *19*, 2560–2574. https://doi.org/10.1101/2022.05.04.22274679.
5. Colom-Cadena, M., Davies, C., Sirisi, S., Lee, J.-E., Simzer, E.M., Tzioras, M., Querol-Vilaseca, M., Sánchez-Aced, É., Chang, Y.Y., Holt, K., et al. (2023). Synaptic oligomeric tau in Alzheimer's disease — A potential culprit in the spread of tau pathology through the brain. Neuron *111*, 2170–2183.e6. https://doi.org/10.1016/j.neuron.2023.04.020.
6. Koffie, R.M., Hashimoto, T., Tai, H.-C., Kay, K.R., Serrano-Pozo, A., Joyner, D., Hou, S., Kopeikina, K.J., Frosch, M.P., Lee, V.M., et al. (2012). Apolipoprotein E4 effects in Alzheimer's disease are mediated by synaptotoxic oligomeric amyloid-β. Brain *135*, 2155–2168. https://doi.org/10.1093/brain/aws127.
7. DeKosky, S.T., and Scheff, S.W. (1990). Synapse loss in frontal cortex biopsies in Alzheimer's disease: correlation with cognitive severity. Ann. Neurol. *27*, 457–464. https://doi.org/10.1002/ana.410270502.
8. Terry, R.D., Masliah, E., Salmon, D.P., Butters, N., DeTeresa, R., Hill, R., Hansen, L.A., and Katzman, R. (1991). Physical basis of cognitive alterations in Alzheimer's disease: synapse loss is the major correlate of cognitive impairment. Ann. Neurol. *30*, 572–580. https://doi.org/10.1002/ana.410300410.
9. Paolicelli, R.C., Bolasco, G., Pagani, F., Maggi, L., Scianni, M., Panzanelli, P., Giustetto, M., Ferreira, T.A., Guiducci, E., Dumas, L., et al. (2011). Synaptic pruning by microglia is necessary for normal brain development. Science *333*, 1456–1458. https://doi.org/10.1126/science.1202529.
10. Schafer, D.P., Lehrman, E.K., Kautzman, A.G., Koyama, R., Mardinly, A.R., Yamasaki, R., Ransohoff, R.M., Greenberg, M.E., Barres, B.A., and Stevens, B. (2012). Microglia sculpt postnatal neural circuits in an activity and complement-dependent manner. Neuron *74*, 691–705. https://doi.org/10.1016/j.neuron.2012.03.026.
11. Cserép, C., Pósfai, B., Lénárt, N., Fekete, R., László, Z.I., Lele, Z., Orsolits, B., Molnár, G., Heindl, S., Schwarcz, A.D., et al. (2020). Microglia monitor and protect neuronal function through specialized somatic purinergic junctions. Science *367*, 528–537. https://doi.org/10.1126/science.aax6752.
12. Lee, J.-H., Kim, J.-Y., Noh, S., Lee, H., Lee, S.Y., Mun, J.Y., Park, H., and Chung, W.-S. (2021). Astrocytes phagocytose adult hippocampal synapses for circuit homeostasis. Nature *590*, 612–617. https://doi.org/10.1038/s41586-020-03060-3.
13. Chung, W.-S., Clarke, L.E., Wang, G.X., Stafford, B.K., Sher, A., Chakraborty, C., Joung, J., Foo, L.C., Thompson, A., Chen, C., et al. (2013). Astrocytes mediate synapse elimination through MEGF10 and MERTK pathways. Nature *504*, 394–400. https://doi.org/10.1038/nature12776.
14. Vainchtein, I.D., Chin, G., Cho, F.S., Kelley, K.W., Miller, J.G., Chien, E.C., Liddelow, S.A., Nguyen, P.T., Nakao-Inoue, H., Dorman, L.C., et al. (2018). Astrocyte-derived interleukin-33 promotes microglial synapse engulfment and neural circuit development. Science *359*, 1269–1273. https://doi.org/10.1126/science.aal3589.
15. Ayata, P., Badimon, A., Strasburger, H.J., Duff, M.K., Montgomery, S.E., Loh, Y.-H.E., Ebert, A., Pimenova, A.A., Ramirez, B.R., Chan, A.T., et al. (2018). Epigenetic regulation of brain region-specific microglia clearance activity. Nat. Neurosci. *21*, 1049–1060. https://doi.org/10.1038/s41593-018-0192-3.
16. Hong, S., Beja-Glasser, V.F., Nfonoyim, B.M., Frouin, A., Li, S., Ramakrishnan, S., Merry, K.M., Shi, Q., Rosenthal, A., Barres, B.A., et al. (2016). Complement and microglia mediate early synapse loss in Alzheimer mouse models. Science *352*, 712–716. https://doi.org/10.1126/science.aad8373.
17. Bie, B., Wu, J., Foss, J.F., and Naguib, M. (2019). Activation of mGluR1 mediates C1q-dependent microglial phagocytosis of glutamatergic synapses in Alzheimer's rodent models. Mol. Neurobiol. *56*, 5568–5585. https://doi.org/10.1007/s12035-019-1467-8.
18. Beckman, D., Ott, S., Donis-Cox, K., Janssen, W.G., Bliss-Moreau, E., Rudebeck, P.H., Baxter, M.G., and Morrison, J.H. (2019). Oligomeric Aβ in the monkey brain impacts synaptic integrity and induces accelerated cortical aging. Proc. Natl. Acad. Sci. USA *116*, 26239–26246. https://doi.org/10.1073/pnas.1902301116.
19. Spurrier, J., Nicholson, L., Fang, X.T., Stoner, A.J., Toyonaga, T., Holden, D., Siegert, T.R., Laird, W., Allnutt, M.A., Chiasseu, M., et al. (2022). Reversal of synapse loss in Alzheimer mouse models by targeting mGluR5 to prevent synaptic tagging by C1Q. Sci. Transl. Med. *14*, eabi8593. https://doi.org/10.1126/scitranslmed.abi8593.
20. Dejanovic, B., Huntley, M.A., De Mazière, A., Meilandt, W.J., Wu, T., Srinivasan, K., Jiang, Z., Gandham, V., Friedman, B.A., Ngu, H., et al. (2018). Changes in the synaptic proteome in tauopathy and rescue of tau-induced synapse loss by C1q antibodies. Neuron *100*, 1322–1336.e7. https://doi.org/10.1016/j.neuron.2018.10.014.
21. Wu, T., Dejanovic, B., Gandham, V.D., Gogineni, A., Edmonds, R., Schauer, S., Srinivasan, K., Huntley, M.A., Wang, Y., Wang, T.-M., et al. (2019). Complement C3 is activated in human AD brain and is required for neurodegeneration in mouse models of amyloidosis and tauopathy. Cell Rep. *28*, 2111–2123.e6. https://doi.org/10.1016/j.celrep.2019.07.060.
22. Shi, Q., Chowdhury, S., Ma, R., Le, K.X., Hong, S., Caldarone, B.J., Stevens, B., and Lemere, C.A. (2017). Complement C3 deficiency protects

(F) Microglia grown from adult mouse brain phagocytose human synapses. Curves from n = 8 mice (mouse as biological replicate) show increased phagocytosis of AD compared with control synapses (ANOVA after linear mixed effects model with synaptoneurosome donor disease status and incubation time as fixed effects and mouse microglia donor as a random effect, effect of disease $F[1,2.88E21] = 102.39$, $p < 0.0001$, effect of incubation time $F[18,\inf] = 98.10$, $p < 0.0001$).
(G) Increased ingestion of AD synaptoneurosomes compared with control ones confirmed by an increased area under the curve in AD (*ANOVA effect of disease status of synapse donor $F[1,14] = 8.96$, $p = 0.01$).

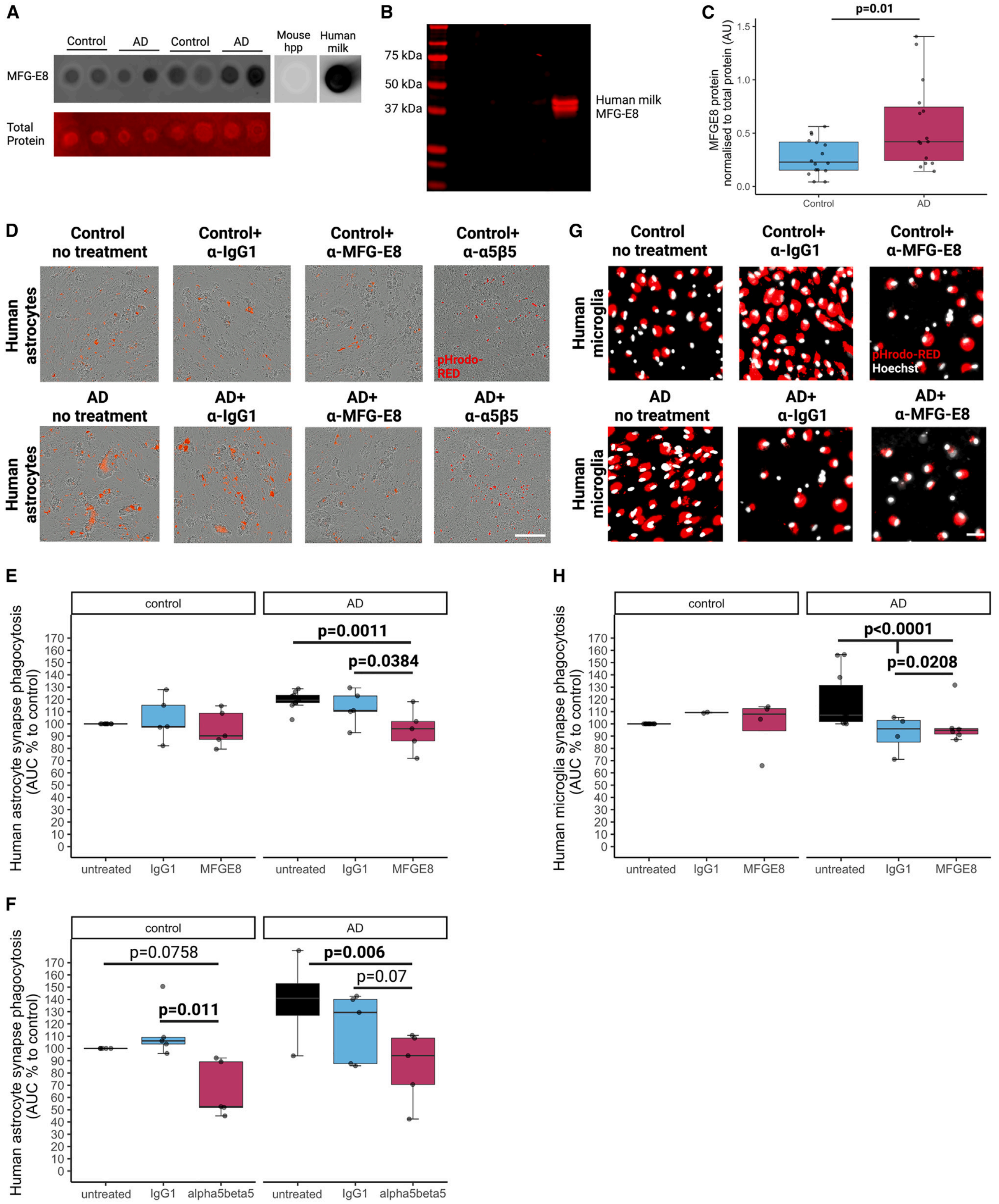

Figure 6. Synaptic MFG-E8 and astrocytic integrin α5β5 modulate phagocytosis of AD synaptoneurosomes

(A) Representative dot blots of aged control and AD-derived synaptoneurosomes probed with MFG-E8. Mouse hippocampus (hpp) was used as a negative control where no MFG-E8 binding is observed and human milk was used as positive control where MFG-E8 is highly expressed.

(legend continued on next page)

against neurodegeneration in aged plaque-rich APP/PS1 mice. Sci. Transl. Med. *9*, eaaf6295. https://doi.org/10.1126/scitranslmed.aaf6295.

23. Tzioras, M., McGeachan, R.I., Durrant, C.S., and Spires-Jones, T.L. (2023). Synaptic degeneration in Alzheimer disease. Nat. Rev. Neurol. *19*, 19–38. https://doi.org/10.1038/s41582-022-00749-z.
24. Saunders, T.S., Pozzolo, F.E., Heslegrave, A., King, D., McGeachan, R.I., Spires-Jones, M.P., Harris, S.E., Ritchie, C., Muniz-Terrera, G., Deary, I.J., et al. (2023). Predictive blood biomarkers and brain changes associated with age-related cognitive decline. Brain Commun. *5*, fcad113. https://doi.org/10.1093/braincomms/fcad113.
25. Bennett, M.L., Bennett, F.C., Liddelow, S.A., Ajami, B., Zamanian, J.L., Fernhoff, N.B., Mulinyawe, S.B., Bohlen, C.J., Adil, A., Tucker, A., et al. (2016). New tools for studying microglia in the mouse and human CNS. Proc. Natl. Acad. Sci. USA *113*, E1738–E1746. https://doi.org/10.1073/pnas.1525528113.
26. Tai, H.-C., Serrano-Pozo, A., Hashimoto, T., Frosch, M.P., Spires-Jones, T.L., and Hyman, B.T. (2012). The synaptic accumulation of hyperphosphorylated tau oligomers in Alzheimer disease is associated with dysfunction of the ubiquitin-proteasome system. Am. J. Pathol. *181*, 1426–1435. https://doi.org/10.1016/j.ajpath.2012.06.033.
27. Graham, L.C., Naldrett, M.J., Kohama, S.G., Smith, C., Lamont, D.J., McColl, B.W., Gillingwater, T.H., Skehel, P., Urbanski, H.F., and Wishart, T.M. (2019). Regional molecular mapping of primate synapses during normal healthy aging. Cell Rep. *27*, 1018–1026.e4. https://doi.org/10.1016/j.celrep.2019.03.096.
28. Byun, Y.G., and Chung, W.-S. (2018). A novel in vitro live-imaging assay of astrocyte-mediated phagocytosis using pH indicator-conjugated synaptosomes. J. Vis. Exp., e56647. https://doi.org/10.3791/56647.
29. Lehrman, E.K., Wilton, D.K., Litvina, E.Y., Welsh, C.A., Chang, S.T., Frouin, A., Walker, A.J., Heller, M.D., Umemori, H., Chen, C., et al. (2018). CD47 protects synapses from excess microglia-mediated pruning during development. Neuron *100*, 120–134.e6. https://doi.org/10.1016/j.neuron.2018.09.017.
30. Grabert, K., Michoel, T., Karavolos, M.H., Clohisey, S., Baillie, J.K., Stevens, M.P., Freeman, T.C., Summers, K.M., and McColl, B.W. (2016). Microglial brain region-dependent diversity and selective regional sensitivities to aging. Nat. Neurosci. *19*, 504–516. https://doi.org/10.1038/nn.4222.
31. Brelstaff, J., Tolkovsky, A.M., Ghetti, B., Goedert, M., and Spillantini, M.G. (2018). Living neurons with tau filaments aberrantly expose phosphatidylserine and are phagocytosed by microglia. Cell Rep. *24*, 1939–1948.e4. https://doi.org/10.1016/j.celrep.2018.07.072.
32. Neniskyte, U., and Brown, G.C. (2013). Lactadherin/MFG-E8 is essential for microglia-mediated neuronal loss and phagoptosis induced by amyloid β. J. Neurochem. *126*, 312–317. https://doi.org/10.1111/jnc.12288.
33. Fricker, M., Oliva-Martín, M.J., and Brown, G.C. (2012). Primary phagocytosis of viable neurons by microglia activated with LPS or Aβ is dependent on calreticulin/LRP phagocytic signalling. J. Neuroinflammation *9*, 196. https://doi.org/10.1186/1742-2094-9-196.
34. Tibaldi, L., Leyman, S., Nicolas, A., Notebaert, S., Dewulf, M., Ngo, T.H., Zuany-Amorim, C., Amzallag, N., Bernard-Pierrot, I., Sastre-Garau, X., et al. (2013). New blocking antibodies impede adhesion, migration and survival of ovarian cancer cells, highlighting MFGE8 as a potential therapeutic target of human ovarian carcinoma. PLoS One *8*, e72708. https://doi.org/10.1371/journal.pone.0072708.
35. Fracassi, A., Marcatti, M., Tumurbaatar, B., Woltjer, R., Moreno, S., and Taglialatela, G. (2023). TREM2-induced activation of microglia contributes to synaptic integrity in cognitively intact aged individuals with Alzheimer's neuropathology. Brain Pathol. *33*, e13108. https://doi.org/10.1111/bpa.13108.

(B) The anti-MFG-E8 antibody was validated by western blot where it strongly binds to human milk at the correct predicted weight of 43 kDa.
(C) Quantification of dot blots in control (n = 16) and Alzheimer's disease (AD) (n = 15) synaptoneurosomes shows significantly higher levels of MFG-E8 in AD samples compared with control ones (ANOVA after linear mixed effects model on Tukey transformed data, $F[1,29] = 7.59$, $p = 0.01$, human brain donor is biological replicate).
(D) Images from phagocytosis assays in human astrocytes treated with pHrodo-Red-labeled human synaptoneurosomes from control and AD brains, illustrating differences in phagocytosis of synapses with pre-treatment of synaptoneurosomes with MFG-E8 antibody, integrin α5β5 antibody, or IgG1 control antibody. Scale bar represents 200 μm.
(E) Area under the curve (AUC % normalized to last control for each experiment) of human astrocytes ingesting control or AD synaptoneurosomes shows statistically significant decrease in phagocytosis of treated with anti-MFG-E8 antibody, but not IgG1, compared with AD untreated synaptoneurosomes and a significant difference between IgG1-treated and MFG-E8-treated synapse phagocytosis (n = 5 independent culture replicates; ANOVA after linear mixed effects model with disease status of synapse donor, time, and treatment as fixed effects and experimental replicate as a random effect shows an effect of disease of synapse donor $F[1,30.2] = 9.674$, $p = 0.004058$; treatment $F[3,32.1] = 237.4$, $p = 2.2 \times 10^{-6}$, and an interaction between disease status of synapse donor and treatment $F[2,30.2] = 4.63$, $p = 0.0176$). Post hoc Tukey corrected tests show in the AD synapse-treated cells that anti-MFG-E8 pre-treatment reduces phagocytosis compared with both no treatment ($p = 0.0011$) and IgG1 treatment ($p = 0.0384$).
(F) Area under the curve (AUC % normalized to last control for each experiment) of human astrocytes ingesting control or AD synaptoneurosomes shows statistically significant decrease in phagocytosis of astrocytes treated with anti-α5β5 antibody, but not IgG1, compared with AD untreated astrocytes and a significant difference between IgG1-treated and anti-α5β5 antibody-treated astrocytes (n = 5 independent replicates; ANOVA after linear mixed effects model with disease status of synapse donor, time, and treatment as fixed effects and experimental replicate as a random effect shows an effect of disease of synapse donor $F[1,20] = 8.71$, $p = 0.00788$ and treatment $F[1,20] = 8.71$, $p = 0.000234$, but not a significant interaction between disease status of synapse donor and treatment $F[2,20] = 2.76$, $p = 0.0872$). Post hoc Tukey corrected tests show in the AD synapse-treated cells that anti-α5β5 pre-treatment reduces phagocytosis compared with no treatment ($p = 0.006$) and non-significant reduction to IgG1 treatment ($p = 0.07$). In control synaptoneurosome-treated cells, there was a non-significant reduction in phagocytosis in anti-α5β5 pre-treatment ($p = 0.0758$) but a significant reduction between IgG1 and anti-α5β5 pre-treatment ($p = 0.011$).
(G) Images from phagocytosis assays in human microglia treated with pHrodo-Red-labeled human synaptoneurosomes from control and AD brains, illustrating differences in phagocytosis of synapses with pre-treatment of synaptoneurosomes with MFG-E8 antibody or IgG1 control antibody. Scale bar represents 20 μm.
(H) Area under the curve (AUC % normalized to last control for each experiment) of human microglia ingesting control or AD synaptoneurosomes show that none of the treatments significantly changes phagocytosis of synapses isolated from control brain. In contrast, both MFG-E8 and IgG1 treatment significantly rescued the amount of microglial phagocytosis back to control levels (ANOVA after linear mixed effects model with disease status of synapse donor, time, treatment, age of microglial donor, brain region donated, and gender of microglial donor as fixed effects and microglial donor ID as a random effect shows significant effects of treatment $F[2,813.97] = 11.69$, $p = 9.84 \times 10^{-6}$ and an interaction between disease status of synapse donor and treatment $F[2,816.83] = 39.19$, $p < 2.2 \times 10^{-16}$). Tukey post hoc tests reveal significant differences in the AD group between no treatment and MFG-E8 antibody treatment ($p < 0.0001$), no treatment and IgG1 treatment ($p < 0.0001$), and between MFG-E8 and IgG1 treatment ($p = 0.0208$). In the control synapse-treated condition, there is a significant difference between untreated and IgG1-treated microglial phagocytosis ($p = 0.036$) with IgG1 treatment causing a slight *increase* in phagocytosis of control synapses. Total n = 11 donors with no treatment, n = 6 MFG-E8 + no treatment, n = 4 IgG1 + MFG-E8 + no treatment.

36. Hesse, R., Hurtado, M.L., Jackson, R.J., Eaton, S.L., Herrmann, A.G., Colom-Cadena, M., Tzioras, M., King, D., Rose, J., Tulloch, J., et al. (2019). Comparative profiling of the synaptic proteome from Alzheimer's disease patients with focus on the APOE genotype. Acta Neuropathol. Commun. *7*, 214. https://doi.org/10.1186/s40478-019-0847-7.

37. Jäkel, S., Agirre, E., Mendanha Falcão, A., van Bruggen, D., Lee, K.W., Knuesel, I., Malhotra, D., Ffrench-Constant, C., Williams, A., and Castelo-Branco, G. (2019). Altered human oligodendrocyte heterogeneity in multiple sclerosis. Nature *566*, 543–547. https://doi.org/10.1038/s41586-019-0903-2.

38. El Hajj, H., Savage, J.C., Bisht, K., Parent, M., Vallières, L., Rivest, S., and Tremblay, M.È. (2019). Ultrastructural evidence of microglial heterogeneity in Alzheimer's disease amyloid pathology. J. Neuroinflammation *16*, 87. https://doi.org/10.1186/s12974-019-1473-9.

39. Gomez-Arboledas, A., Davila, J.C., Sanchez-Mejias, E., Navarro, V., Nuñez-Diaz, C., Sanchez-Varo, R., Sanchez-Mico, M.V., Trujillo-Estrada, L., Fernandez-Valenzuela, J.J., Vizuete, M., et al. (2018). Phagocytic clearance of presynaptic dystrophies by reactive astrocytes in Alzheimer's disease. Glia *66*, 637–653. https://doi.org/10.1002/glia.23270.

40. Sanchez-Mico, M.V., Jimenez, S., Gomez-Arboledas, A., Muñoz-Castro, C., Romero-Molina, C., Navarro, V., Sanchez-Mejias, E., Nuñez-Diaz, C., Sanchez-Varo, R., Galea, E., et al. (2021). Amyloid-β impairs the phagocytosis of dystrophic synapses by astrocytes in Alzheimer's disease. Glia *69*, 997–1011. https://doi.org/10.1002/glia.23943.

41. Sellgren, C.M., Gracias, J., Watmuff, B., Biag, J.D., Thanos, J.M., Whittredge, P.B., Fu, T., Worringer, K., Brown, H.E., Wang, J., et al. (2019). Increased synapse elimination by microglia in schizophrenia patient-derived models of synaptic pruning. Nat. Neurosci. *22*, 374–385. https://doi.org/10.1038/s41593-018-0334-7.

42. Tzioras, M., Stevenson, A.J., Boche, D., and Spires-Jones, T.L. (2021). Microglial contribution to synaptic uptake in the prefrontal cortex in schizophrenia. Neuropathol. Appl. Neurobiol. *47*, 346–351. https://doi.org/10.1111/nan.12660.

43. Paolicelli, R.C., Jawaid, A., Henstridge, C.M., Valeri, A., Merlini, M., Robinson, J.L., Lee, E.B., Rose, J., Appel, S., Lee, V.M.-Y., et al. (2017). TDP-43 depletion in microglia promotes amyloid clearance but also induces synapse loss. Neuron *95*, 297–308.e6. https://doi.org/10.1016/j.neuron.2017.05.037.

44. Del-Aguila, J.L., Li, Z., Dube, U., Mihindukulasuriya, K.A., Budde, J.P., Fernandez, M.V., Ibanez, L., Bradley, J., Wang, F., Bergmann, K., et al. (2019). A single-nuclei RNA sequencing study of Mendelian and sporadic AD in the human brain. Alzheimer's Res. Ther. *11*, 71. https://doi.org/10.1186/s13195-019-0524-x.

45. Lau, S.-F., Cao, H., Fu, A.K.Y., and Ip, N.Y. (2020). Single-nucleus transcriptome analysis reveals dysregulation of angiogenic endothelial cells and neuroprotective glia in Alzheimer's disease. Proc. Natl. Acad. Sci. USA *117*, 25800–25809. https://doi.org/10.1073/pnas.2008762117.

46. Sokolova, D., Childs, T., and Hong, S. (2021). Insight into the role of phosphatidylserine in complement-mediated synapse loss in Alzheimer's disease. Fac. Rev. *10*, 19.

47. Thrupp, N., Sala Frigerio, C., Wolfs, L., Skene, N.G., Fattorelli, N., Poovathingal, S., Fourne, Y., Matthews, P.M., Theys, T., Mancuso, R., et al. (2020). Single-nucleus RNA-seq is not suitable for detection of microglial activation genes in humans. Cell Rep. *32*, 108189. https://doi.org/10.1016/j.celrep.2020.108189.

48. Olmos-Alonso, A., Schetters, S.T.T., Sri, S., Askew, K., Mancuso, R., Vargas-Caballero, M., Holscher, C., Perry, V.H., and Gomez-Nicola, D. (2016). Pharmacological targeting of CSF1R inhibits microglial proliferation and prevents the progression of Alzheimer's-like pathology. Brain *139*, 891–907. https://doi.org/10.1093/brain/awv379.

49. Grabert, K., and McColl, B.W. (2018). Isolation and phenotyping of adult mouse microglial cells. Methods Mol. Biol. *1784*, 77–86. https://doi.org/10.1007/978-1-4939-7837-3_7.

50. Blain, M., Miron, V., Lambert, C., Darlington, P.J., Cui, Q.-L., Saikali, P., Wee Yong, V., and Ante, J.P. (2010). Isolation and culture of primary human CNS neural cells. In Protocols for Neural Cell Culture.

51. Hasel, P., Dando, O., Jiwaji, Z., Baxter, P., Todd, A.C., Heron, S., Márkus, N.M., McQueen, J., Hampton, D.W., Torvell, M., et al. (2017). Neurons and neuronal activity control gene expression in astrocytes to regulate their development and metabolism. Nat. Commun. *8*, 15132. https://doi.org/10.1038/ncomms15132.

52. Baxter, P.S., Dando, O., Emelianova, K., He, X., McKay, S., Hardingham, G.E., and Qiu, J. (2021). Microglial identity and inflammatory responses are controlled by the combined effects of neurons and astrocytes. Cell Rep. *34*, 108882. https://doi.org/10.1016/j.celrep.2021.108882.

53. Zhang, Y., Sloan, S.A., Clarke, L.E., Caneda, C., Plaza, C.A., Blumenthal, P.D., Vogel, H., Steinberg, G.K., Edwards, M.S.B., Li, G., et al. (2016). Purification and characterization of progenitor and mature human astrocytes reveals transcriptional and functional differences with mouse. Neuron *89*, 37–53. https://doi.org/10.1016/j.neuron.2015.11.013.

54. Team, Rs (2022). RStudio: Integrated Development Environment for R.

55. Spires-Jones, T.L. (2022). Using R to improve rigour and transparency in translational neuroscience—or is it just a rabbit hole? Brain Commun. *4*, fcab290. https://doi.org/10.1093/braincomms/fcab290.

CellPress
OPEN ACCESS

STAR★METHODS

KEY RESOURCES TABLE

REAGENT or RESOURCE	SOURCE	IDENTIFIER
**Antibodies**		
Mouse anti CD68 (used 1:100)	Agilent M0876	RRID:AB_2074844
Rabbit anti P2RY12 (used 1:500)	Sigma-Aldrich HPA014518	RRID:AB_2669027
Rabbit anti Synapsin 1 (used 1:750)	Merck Millipore AB1543P	RRID:AB_90757
Mouse anti synaptophysin (used 1:500)	Abcam ab8049	RRID:AB_2198854
Rabbit anti synaptophysin, direct labeled Alexa Fluor™ 488 (used 1:500)	Abcam ab196379	RRID:AB_2922671
Guinea pig anti PSD-95 (used 1:500)	Synaptic systems 124 014	RRID:AB_2619800
Goat anti Iba1 (used 1:500)	Abcam ab5076	RRID:AB_91676
Rabbit anti TMEM119 (used 1:500)	Abcam ab185333	RRID:AB_2687894
Rabbit anti AW7 (used 1:5,000)	Provided by Dominic Walsh	RRID:AB_2313982
Chicken anti GFAP (used 1:2,000/1:3,000)	Abcam ab53554	RRID:AB_880202
Rabbit anti GAD65/67 (used 1:500)	Abcam ab55412	RRID:AB_880148
Rabbit anti Histone H3 (used 1:1000)	Abcam ab1791	RRID:AB_302613
Rabbit anti PSD-95 (used 1:1000)	Cell Signaling D27E11	RRID:AB_2292883
Mouse anti pan-neurofilament (SMI-312) (used 1:500)	BioLegend 837904	RRID:AB_2566782
Sheep anti MFG-E8 (used 1:500)	R&D Systems AF2767	RRID:AB_10889829
Mouse anti MFG-E8 (used at 10 μg/mL)	Creative Biolabs FAMAB-0225CQ-LowE	N/A
Mouse anti IgG1 (used at 10 μg/mL)	Creative Biolabs MOB-065CQ	N/A
Mouse anti integrin $\alpha 5 \beta 5$ (used at 2.5 μg/mL)	R&D Systems MAB2528	RRID:AB_2280706
Mouse anti CD11b-Alexa Fluor™ 594 (used 5 μg/mL)	Biolegend 301340	RRID:AB_2563208
Mouse anti CD45-APC (used 1:20)	Biolegend 304011	RRID:AB_314399
Rat anti CX3CR1-PECy7 (used 0.1 μg/mL)	Biolegend 341611	RRID:AB_2297689
Rat anti CD16/32 (Fc block against mouse) (used 1:1000)	Biolegend 101302	RRID:AB_312801
Human TruStain FcX™ Fc block (Fc block against human) (used 1:1000)	Biolegend 422302	RRID:AB_2818986
Donkey anti mouse Alexa Fluor™ 594 (used 1:500)	Thermo Fisher Scientific A-21203	RRID:AB_141633
Donkey anti rabbit Alexa Fluor™ 488 (used 1:500)	Thermo Fisher Scientific A-21206	RRID:AB_2535792
Donkey anti rabbit Alexa Fluor™ 594 (used 1:500)	Thermo Fisher Scientific A-21207	RRID:AB_141637
Donkey anti rabbit Alexa Fluor™ 647 (used 1:500)	Thermo Fisher Scientific A-31573	RRID:AB_2536183
Donkey anti goat Alexa Fluor™ 647 (used 1:500)	Thermo Fisher Scientific A32849	RRID:AB_2762840
Goat anti chicken Alexa Fluor™ 647 (used 1:500)	Thermo Fisher Scientific A32933	RRID:AB_2762845
Donkey anti chicken Alexa Fluor™ 405 (used 1:500)	Jackson ImmunoResearch 103-475-155	RRID:AB_2337389
Goat anti Guinea pig Alexa Fluor™ 594 (used 1:500)	Thermo Fisher Scientific A-11076	RRID:AB_141930
Goat anti mouse IgM Alexa Fluor™ 594 (used 1:500)	Thermo Fisher Scientific A-21044	RRID:AB_2535713
Rabbit anti-goat HRP (used 1:5000)	Abcam ab6741	RRID:AB_955424
IRDye 680RD Donkey anti-mouse (used 1:5000)	LI-COR 925-68072	RRID:AB_2814912
IRDye 800CW Donkey anti-rabbit (1:5000)	LI-COR 925-32213	RRID:AB_2715510
**Biological samples**		
Human postmortem brain samples (paraffin embedded and fresh-frozen) and live brain samples for cell isolation	MRC Edinburgh Brain Bank	See Tables S1 and S2 for all sample info

(*Continued on next page*)

Continued		
REAGENT or RESOURCE	SOURCE	IDENTIFIER
Chemicals, peptides, and recombinant proteins		
Citrate buffer pH 6	Vector labs	H3300
Autofluorescence eliminator reagent	Merck	2160
TrueVIEW ® Autofluorescence Quenching Kit	Vector labs	SP-8400-15
Triton X-100	Sigma-Aldrich	T8787-100ML
Normal donkey serum	Sigma-Aldrich	D96663
Normal goat serum	Sigma-Aldrich	S26-100ML
DAPI	Sigma-Aldrich	D9542-10MG
Hoechst	ThermoFisher Scientific	H3570
Cellmask deep red	Life technologies	C10046
Thioflavin S	Sigma-Aldrich	T1892
Ponceau S	Sigma-Aldrich	P7170-1L
Immumount	ThermoFisher Scientific	9990402
pHrodo^{TM} Red-SE	ThermoFisher Scientific	P36600
NuPAGE^{TM} buffer	ThermoFisher Scientific	NP0002
Odyssey blocking buffer	LI-COR	927–40000
Protease inhibitors	Merck	11836170001
Phosphatase inhibitors	Merck	524629-1SET
Protease inhibitor cocktail EDTA-free	ThermoFisher Scientific	78447
Western blot molecular weight marker	LI-COR	928–40000
DMEM + GlutaMAX	ThermoFisher Scientific	31966–021
DMEM/F-12	ThermoFisher Scientific	11320033
Astrocyte medium	Caltag Medsystems	SC-1801
Percoll	GE Healthcare	GE17-0891-01
Human mCSF1	R&D Systems	216-MC-010/CF
Mouse mCSF1	R&D Systems	416-ML-010/CF
Recombinant human TGFβ-1	Miltenyi	170-076-166
Low endotoxin BSA	Sigma-Aldrich	A8806
Fetal bovine serum (heat inactivated)	ThermoFisher Scientific	10500064
Penicillin/streptomycin	ThermoFisher Scientific	15070063
Cytochalasin D	Sigma-Aldrich	C8273
K777	Adipogen	AG-CR1-0158
IL-34	Peprotech	20034205
BDNF	Peprotech	45002100
Critical commercial assays		
Neural Tissue Dissociation Kit (P)	Miltenyi	130-092-628
Micro BCA kit	ThermoFisher Scientific	23235
CD11b microbeads	Miltenyi	130-093-634
SuperSignal^{TM} West Dura Extended Duration Substrate	ThermoFisher Scientific	34075
Deposited data		
All spreadsheets and statistical analysis files can be found at https://doi.org/10.7488/ds/7492	N/A	N/A
All image analysis scripts are available on Github at https://github.com/Spires-Jones-Lab.	N/A	N/A
Experimental models: Cell/mouse lines		
Primary human astrocytes	Caltag Medsystems (Sciencell)	SC-1800
BV2 cells		
C57Bl/6J mice	Charles River Laboratories	000664

(*Continued on next page*)

CellPress
OPEN ACCESS

Continued

REAGENT or RESOURCE	SOURCE	IDENTIFIER
Software and algorithms		
R Studio Version 2022.12.0 + 353	GitHub	https://github.com/rstudio/rstudio
R version 4.1.2 GUI 1.77 High Sierra build	https://www.r-project.org/	R 4.1.2 GUI 1.77 High Sierra build
Color scheme for R Studio graphs:	GitHub	https://github.com/asteves/tayloRswift
Prism 9	GraphPad	Prism 9
FIJI Version 2.0.0-rc-54/1.52t	GitHub	FIJI Version 2.0.0-rc-54/1.52t
MATLAB Version R2022a	Mathworks	Version R2022a
Docker Version 4.10.1 (82475)	Docker	Version 4.10.1 (82475)

RESOURCE AVAILABILITY

Lead contact

Further information and requests for resources and reagents should be directed to and will be fulfilled by the lead contact, Tara Spires-Jones (tara.spires-jones@ed.ac.uk).

Materials availability

This study did not generate new unique reagents.

Data and code availability

All spreadsheets of analyzed data and statistical analysis scripts are available on the University of Edinburgh DataShare repository https://doi.org/10.7488/ds/7492. All image analysis scripts are available on Github at https://github.com/Spires-Jones-Lab. Raw images from analyses are available upon request from the lead contact. Any additional information required to analyze the data reported in thus paper is available from the lead contact upon request.

EXPERIMENTAL MODEL AND SUBJECT DETAILS

Animals

Microglial isolation experiments were performed using 12-week-old male C57Bl/6J mice (Charles River Laboratories). Primary astrocyte isolations were performed on E17.5 CD1 mouse embryos of both sexes. Mice were maintained under a standard 12 h light/dark cycle and provided with *ad libitum* access to food and water. Mice were housed in groups of up to five mice and were acclimatized for a minimum of 1 week prior to procedures. All experiments were conducted under the UK Home Office Animals (Scientific Procedures) Act 1986, in agreement with local ethical and veterinary approval (Biomedical Research Resources, University of Edinburgh).

Human postmortem tissue

All tissue was provided by the MRC Edinburgh Brain Bank, following all appropriate ethical approval and informed consent of the donors pre-mortem and their families postmortem. For paraffin embedding, tissue was dehydrated via increasing ethanol solutions, fixed in formalin, and baked in paraffin-embedded blocks. Tissue from the inferior temporal lobe (Brodmann area 20/21) and primary visual cortex (Brodmann area 17) was cut using a microtome at 4μm thickness and mounted on glass slides for use in immunohistochemistry. The locations of these areas in the brain are visualised in Figure 1. AD cases were cross-checked neuropathologically and were confirmed to be Braak Stages V-VI. In one case (BBN: 24527) no plaques were measured in BA17 and was excluded as a whole from the near plaque analysis. Case BBN31495 has a Braak Stage of VI but has been cognitively tested upon 3 waves and was cognitively unimpaired. Data about subjects included in the study are found in Table S1.

Use of human tissue for postmortem studies has been reviewed and approved by the Edinburgh Brain Bank ethics committee and the ACCORD medical research ethics committee, AMREC (approval number 15-HV-016; ACCORD is the Academic and Clinical Central Office for Research and Development, a joint office of the University of Edinburgh and NHS Lothian). The Edinburgh Brain Bank is a Medical Research Council funded facility with research ethics committee (REC) approval (11/ES/0022).

BV2 microglia phagocytosis assay

Phagocytosis assays were optimised using the BV2 immortalised murine microglia cell line. BV2 microglia were cultured in DMEM + GlutaMAX (ThermoFisher Scientific, 31966-021) and were supplemented with 10% fetal bovine serum (FBS, ThermoFisher Scientific) and 1% penicillin/streptomycin (PenStrep, ThermoFisher Scientific). Cells were grown in a humidity-controlled incubator at 37°C with 5% CO_2. One day prior to the phagocytosis assay, BV2 microglia were seeded in a 96-well flat bottom plate (ThermoFisher Scientific, 165305) at a density of 12,500 cells/well. Cells were stained with Hoechst (2 μg/mL, ThermoFisher, H3570) to visualise nuclei and

cytochalasin D treated cells (10 μM, Sigma-Aldrich, C8273) were used as negative controls. BV2 cells were challenged with pHrodo-tagged synaptoneurosomes, and immediately taken to an ImageExpress high-throughput microscope (Molecular Devices) for live-imaging using a 20x air objective (37°C, 5% CO_2). Images were taken every 5–10 min for 3 h, using the same settings for all imaging sessions. All conditions were repeated in triplicate with 4 fields of view per well. For analysis, MetaXpress 6 (Molecular Devices) software was used to automatically calculate the number of cells per field of view and detect fluorescence around cells by thresholding. A phagocytosis index was calculated by normalizing the number of cells phagocytosing to total number of cells in an automated and unbiased way. The same exposure settings were used for each set of experiments. Videos taken with phase contrast were recorded on a Zeiss Observer Z1 microscope using 20x air objective (37°C, 5% CO_2), with images taken every 5 min.

Primary mouse microglia

Primary adult mouse microglia were isolated and cultured as described previously.[49] Brains from 12-week-old male C57/Bl6 (Charles-River) mice were isolated by terminally anesthetizing with 3% isoflurane (33.3% O_2 and 66.6% N_2O) and transcardial perfusion with ice-cold 0.9% NaCl. Brains were immediately placed into ice-cold HBSS (ThermoFisher Scientific) and minced using a 22A scalpel before centrifugation (300 x g, 2 min) and digestion using the MACS Neural Dissociation Kit (Miltenyi) according to manufacturer's instructions. Briefly, brain tissue was incubated in enzyme P (50 μL/brain) diluted in buffer X (1900 μL/brain) for 15 min at 37°C under gentle rotation before addition of enzyme A (10 μL/brain) in buffer Y (20 μL/brain) and further incubation for 20 min at 37°C under gentle rotation. Following digest tissue was dissociated mechanically using a Dounce homogenizer (loose Pestle, 20 passes) on ice and centrifuged (400 x g, 5 min at 4°C). To remove myelin, tissue was resuspended in 35% isotonic Percoll (GE Healthcare, GE17-0891-01), overlaid with 2 mL HBSS, and centrifuged (800 x g, 40 min, 4°C). Following centrifugation, the supernatant and myelin layers were discarded and the pellet resuspended in MACS buffer (PBS, 0.5% low endotoxin BSA (Sigma-Aldrich), 2 mM EDTA, 90 μL/brain). Anti-CD11b microbeads (Miltneyi) were added (10 μL/brain) and the suspension incubated for 15 min at 4°C. before running through pre-rinsed (MACS buffer) LS columns attached to a magnet (Miltenyi). After washing with 12 mL MACS buffer, columns were removed from the magnet and cells retained (microglia) were flushed in 5 mL MACS buffer. Microglia were resuspended in DMEM/F-12 (ThermoFisher Scientific) supplemented with 1% PenStrep, 10% FBS, 50 ng/mL rhTGFβ-1 (Miltenyi), 10 ng/μL mCSF1 (R&D Systems). Microglia were counted using a haemocytometer and plated out at 40,000 cells/well onto a black-walled, optical bottom 96-well plate (ThermoFisher Scientific) coated with poly-L-lysine. Cells were cultured for 7 days with a half media change on day 3. Phagocytosis assay was performed and analyzed as described below in methods details with 9 fields of view per well.

Primary human (GBM) microglia isolation

Human microglial isolations were performed as described previously.[50] Use of human brain tumor and peri-tumoral tissue resected at surgery for research was approved by National Health Service Lothian under protocol number LREC 15/ES/0094 issued to P.M.B. with full informed consent. For isolation of human microglia, the same protocol was used as the mice (CD11b beads) with a few adjustments detailed as follows. For all steps until the Percoll gradient, RPMI (ThermoFisher, 11875093) with 3% FBS +2 mM EDTA was used instead of HBSS. Additionally, as the tissue was not perfused, an additional treatment with red blood cell lysis buffer (Biolegend, 420301) was carried out following the Percoll gradient step. Microglia were counted using a haemocytometer and plated out at 40,000 cells/well onto a black-walled, optical bottom 96-well plate (ThermoFisher Scientific) coated with poly-L-lysine. Cells were cultured in DMEM/F-12 (ThermoFisher Scientific) supplemented with 1% PenStrep, 10% FBS, 50 ng/mL rhTGFβ-1 (Miltenyi), 10 ng/μL mCSF1 (R&D Systems) for 7 days with a half media change on day 3. Phagocytosis assay was performed and analyzed as described above with 9 fields of view per well. Exclusion criteria for a case/donor was poor viability of the cells at the end of the experiment.

Primary human (epilepsy case) microglia isolation

Use of human temporal lobe resections for research was approved by National Health Service Lothian under protocol number 2017/0125/SR/720 issued to V.E.M. with full informed consent. The protocol for isolating primary human microglia was adapted from a previously established one.[50] Fresh brain tissue was donated from a 23-year-old male undergoing epilepsy surgery. Resected brain specimen came from healthy tissue of the temporal lobe. Briefly, blood was removed by multiple washes of PBS followed by treatment with 0.25% trypsin and 100ug/mL DNAse in PBS for 30 min at 37°C, with gentle rotation. Trypsin was then deactivated with 10% FCS. Samples were centrifuged at 1,200 RPM for 10 min (high brakes) at 4°C and the supernatant was discarded followed by addition of PBS and Percoll (GE Healthcare) with further ultra-centrifugation at 15,000 RPM for 30 min at 4°C (no brakes). The myelin layer was aspirated off and cell layer was transferred in a clean tube, leaving behind a layer of red blood cells. The transferred cells were topped-up with PBS and centrifuged again at 1,200 RPM for 10 min (high brakes) at 4°C and resuspended in warm media containing 5% FCS and 0.1% glucose. Subsequently, isolated mixed cells were cultured in T12.5 flasks for 3 days. Three days later, microglia were trypsinized and collected from the flasks, following centrifugation to yield the microglia cell pellet. After the isolation, microglia were counted using a haemocytometer and plated out at 40,000 cells/well onto a black-walled, optical bottom 96-well plate (ThermoFisher Scientific) coated with poly-L-lysine, and were allowed to rest for 5 days prior to phagocytosis assays. Phagocytosis assay was performed and analyzed as described above with 9 fields of view per well.

Human pluripotent stem cell derived microglia cultures

Human pluripotent stem cells were routinely maintained in mTESR media in GELTREX coated dishes. Pluripotent stem cells were differentiated to primitive macrophages using a modified protocol from Takata et al. (2017) [54]. Briefly, on day 0, PSC clumps were seeded on GELTREX coated dishes in mTESR media at 100,000 cells per $10cm^2$. Stempro-34 media (ThermoFisher Scientific, 10639011) was supplemented with 5 μM ascorbic acid (Sigma-Aldrich, A5960), Glutamax (ThermoFisher Scientific, 35050038), penicillin and streptomycin (ThermoFisher Scientific, 15140122), apo transferrin (Sigma-Aldrich, T1147), monothioglycerol (MTG) (Sigma-Aldrich, M6145-25mL) and mixed fresh with growth factor combinations for every timepoint of the protocol. Floating cells were collected from day 8 and reseeded in fresh media after centrifugation. On day 16, media was switched to serum-free defined media consisting of IMDM (ThermoFisher Scientific, 31980030) and F12 (3:1 ratio) (ThermoFisher Scientific, 11765054) supplemented with 0.5% N2 (W-MRC CSCI in house) and 1% B27 supplement (ThermoFisher Scientific, 17504044) and BSA fraction V (0.1% g/100mL), penicillin and streptomycin and 50 ng/mL M-CSF (Miltenyi biotech, 130-096-493). Floating primitive macrophages were collected between day 28 and 35 and analyzed for CD11b (CD11b-A594, Biolegend, 301340), CD45 (CD45-APC, Biolegend, 304011), CX3CR1 (CX3CR1-PECy7, Biolegend, 341611) expression by flow cytometry. Primitive macrophages were seeded on isogenic human cortical neuron cultures at 1:5 macrophage to neuron ratio in N2B27 co-culture media supplemented with 50 ng/mL IL34 (Peprotech, 20034205), 10 ng/mL M-CSF and 10 ng/mL BDNF (Peprotech, 45002100). Half the media was replaced with fresh media every 2–3 days. Microglia cells were collected from co-cultures after at least 2 weeks of coculture by magnetic immunolabelled sorting for CD11b+ cells (CD11b microbeads, Miltenyi biotech, 130-093-634). Purified microglia were replated on PDL coated Ibidi 8u slides in neuron conditioned co-culture medium supplemented with fresh cytokines with 5×10^4 cells per well ($1cm^2$). Cells were maintained for less than a week before imaging. Cellmask deep red (Life technologies, C10046) was used as contrast membrane labeling for 30 min before imaging. Imaging was done on Zeiss 710 with Ibidi incubator unit taking images from 10 regions of interest per condition. Image-series were analyzed on Volocity software to measure pHrodo red intensity changes in cellmask deep red positive cells. Cell numbers measured for each cell line per condition.

Primary mouse astrocyte isolation

Astrocyte isolation and culturing has been previously described.[51] Glia cultures were achieved by passaging a mixed neuron/glia culture with trypsin consequently removing neurons. All reagents were purchased from Merk unless otherwise stated. Briefly, E17.5 mouse CD1 embryos were decapitated in accordance with schedule 1 of UK home office guidelines for humane killing of animals. The cortices were removed in a dissociation medium (81.8 mM Na_2SO_4, 30 mM K_2SO_4, 5.84 mM $MgCl_2$, 0.252 mM CaCl2, 1 mM HEPES, 20 mM D-glucose, 1 mM kynurenic acid, 0.001% Phenol Red) and then enzymatically digested with papain at 36,000 USP units/mL for 40 min. The cortices were then washed twice with dissociation medium followed by twice with plating media (DMEM+10% FBS+1X antibiotic-antimycotic agent (all Thermofisher)). Cortices were homogenized using a 5mL pipette by sequential suction/expulsion and plated a density of 2 cortices/T75 flask in plating medium. After 6–7 days *in vitro*, the mixed neuron/glia culture was trypsinized, centrifuged at 150 x g for 5 min and the pellet re-suspended in plating medium. 1/3 of the total cell suspension was plated into a new T75 which reached maximum confluency 4–5 days later. This cell population now predominately contains astrocytes with a smaller proportion of microglia which can be detached by shaking. Plating of astrocytes was done similar to microglia, as described above.

Primary human astrocyte line

Human astrocytes were purchased by Caltag Medsystems (SC-1800) and cultured in poly-D-lysine coated 96-well plates with Astrocyte medium (Caltag Medsystems, SC-1801), as described previously.[52] In previous work, the characterization was confirmed by comparing genome-wide transcriptomic data and comparing it to published data relating to acutely purified human astrocytes, neurons, microglia and oligodendrocytes.[52,53] The expression of genes in primary human astrocytes (co-cultured with mouse neurons and rat microglia) significantly correlated with the expression in acutely isolated human astrocytes (*$p > 0.0001$). Furthermore, genes predominantly expressed in acutely sorted human astrocytes compared to neurons, microglia or oligodendrocytes, were also significantly enriched in primary human astrocytes when compared to the other cell-types (*$p > 0.002$).

METHOD DETAILS

Immunohistochemistry for paraffin embedded human tissue

Paraffin-embedded sections were provided by the Sudden Death MRC Edinburgh Brain Bank and. Tissue was resected from postmortem brains and dehydrated with ethanol, prior to paraffin-embedding. Sections were cut using a microtome at 4 μm thickness and provided upon a justified tissue request. Slides with embedded tissue were dewaxed in xylene for 6 min, followed by rehydration using descending ethanol-to-water solutions: 100% ethanol, 90% ethanol, 70% ethanol, 50% ethanol, 100% water, of 3 min each. For antigen retrieval, samples were pressure cooked for 3 min at the steam setting in citrate buffer, pH 6 (Vector labs, H3300). Specifically, citric acid concentrate was diluted from 100x stock in de-ionised water for use, and made fresh each time. Slides were allowed to cool down under running water, and then immersed in 70% ethanol for 5 min. Slides were incubated with an autofluorescence eliminator reagent (Merck, 2160) for 5 min and washed with 70% ethanol, followed by two 5-min washes using PBS-0.3% Triton X-100 (Sigma-Aldrich, T8787-100ML), and one wash with 1x PBS (Thermo Fisher, 70011036). Noise from red blood

cells was eliminated using the Vector TrueVIEW Autofluorescence Quenching Kit (SP-8400-15). Using a wax pen (Vector labs, H4000), the tissue was outlined and incubated with blocking solution for 1 h. Blocking solutions consisted of 0.3% Triton X-100 and either 10% normal donkey serum (Sigma-Aldrich, D96663) or 5% normal donkey serum and 5% normal goat serum (Sigma-Aldrich, S26-100ML). Primary antibodies were diluted appropriately (key resources table) at a final volume of 500μL per slide, and were allowed to incubate overnight (14–16 h) in the cold room, at 4-6°C, in a humid chamber using wet paper towel in the staining box. On the second day of staining, slides were washed once in PBS-0.3% Triton X-100, followed by two 5-min washes in PBS. Secondary antibodies were then applied at a volume of 500μL per slide (key resources table). All cross-adsorbed secondary antibodies were made-up to a final concentration of 1:500 (4 μg/mL) in PBS, and applied for 1 h at room temperature. Nuclei were counterstained with DAPI (1 μg/mL) (D9542-10MG, Sigma-Aldrich). For Thioflavin S (Sigma-Aldrich, T1892), slides were dipped in 0.001% Thioflavin S, made in 50% ethanol, for 8 min and differentiated in 80% ethanol for 1 min. In the end, one drop of Immumount (Thermo Fisher, 9990402) was added per slide to allow for coverslip adherence. Coverslips size was No 1.5, corresponding to 22 × 40mm (VWR, 631-0136). Coverslips were pressed gently to remove excess mounting media and remove bubbles, and were allowed to dry at room temperature for at least 1 h. Slides were kept in the fridge for long-term storage and were allowed to reach room temperature prior to imaging.

Confocal microscopy and image analysis

Slides were imaged on a confocal microscope (Leica TCS8) with a 63x oil immersion objective. Laser and detector settings were kept constant between samples. During imaging and analysis, the experimenter was blinded to brain area and disease status. Twenty images from the gray matter were taken per case, sampling randomly through all six cortical layers. The synaptic stain was used to confidently separate gray matter versus white matter. The resolution of the confocal microscopy was 0.18 μm × 0.18 μm x 0.3μm (xyz). Image stacks were segmented in 3D in MATLAB under the auto-local threshold function with custom made scripts. For the CD68 stain, the MATLAB segmentation setting were: window size = 70, Factor C = 0.2, method = mean, no minimum size selected. For the synapsin I stain the settings were: window size = 10, Factor C = 1, method = mean, no minimum size selected. For the GFAP stain, the MATLAB segmentation setting were: window size = 70, Factor C = 0.15, method = mean, no minimum size selected. The same segmentation parameters were used for all images to keep the final outputs similar and not influence the final burdens between cases. Once the segmentation was completed, the images were processed with a custom Python script in order to obtain their volumetric measurements (i.e., density of objects/mm^3 of tissue). This provided density measurements for stained markers, like the total objects of CD68-positive staining per image slice in a z stack. This allowed further analysis on Python for calculating the area of ormalizedion between CD68/GFAP and SynI objects in 3D. To allow colocalization, two objects need to overlap at least 25% of their whole structure. Images were processed again with FIJI in order to add all segmented slices in their respective stacks in order to calculate the volume occupied by all of the staining in the stack. Each stack was normalized to the volume of the entire stack (different stacks have different amounts of slices, so the final volume differs). Segmentation scripts can be found in the following link: https://github.com/lewiswilkins/Array-Tomography-Tool.

Airyscan confocal microscopy

Images were acquired using a Zeiss LSM880 with Airyscan (Carl Zeiss Ltd, Cambridge, UK) point scanning confocal, fitted to an Axio Examiner Z1 microscope stand (Carl Zeiss Ltd, Cambridge, UK), running Zen Black 2.3. A 63 × 1.4 NA oil immersion objective was used (Carl Zeiss Ltd, Cambridge, UK), with the Airyscan detector set to SR-mode. Images were acquired with optimal pixel size and z-interval.

Synaptoneurosome preparation

Preparation of synaptoneurosomes was performed as described previously.[26] Snap-frozen human tissue of 300-500 mg from BA38 (temporal cortex) was homogenised using a Dounce homogeniser with 1mL of a protease inhibitor buffer, termed here Buffer A. Buffer A consists of 25 mM HEPES, 120 mM NaCl, 5 mM KCl, 1 mM $MgCl_2$, 2 mM $CaCl_2$, protease inhibitors (Merck, 11836170001) and phosphatase inhibitors (Merck, 524629-1SET) made up in sterile water, and was prepared fresh each day. The Dounce homogeniser and Buffer A were kept ice-cold throughout the procedure to avoid further degradation. We allowed 10 passes for full homogenization but minimise cellular disruption that would lead to an impure final fraction. Once homogenised, the homogenate was aspirated in a 1mL syringe and passed through an 80-micron filter (Merck, NY8002500) to remove debris and yielded the total homogenate (TH). The filter was pre-washed with 1mL of Buffer A to maximise yield. A sample of the TH was snap-frozen on dry ice for Western blot analysis, and the rest was split in half for Western blot analysis and the phagocytosis assays. A subsequent filtration took place using a 5-micron filter (Merck, SLSV025LS) followed by centrifugation at 1000 x g for 7 min to yield the synaptoneurosome (SN) pellet. In this step, extra care was taken to slowly pass the homogenate through the filter in order to prevent the filter breaking. The filter was pre-washed with 1mL of Buffer A to maximise yield. The pellet was washed with Buffer A and pelleted down again to ensure purity. Pellets were snap frozen on dry ice and stored at −80°C for long-term storage.

Synaptosome preparation

Synaptosome preparations were made as described previously.[27] Frozen human brains from BA38 and BA41/42 were homogenised in ice-cold Dounce homogenisers (10 passes) in 1mL of 0.32 M sucrose solution (0.32 M sucrose, 1 mM EDTA, 5 mM Tris-HCl,

protease inhibitors (Merck, 11836170001) and phosphatase inhibitors (Merck, 524629-1SET), pH 7.4). The homogenate was spun at 900 x g for 4 min at 4°C and the supernatant was collected. The supernatant was spun again at 20,000 x g for 20 min at 4°C and the pellet was kept as the synaptosome fraction. Synaptosome pellets were snap-frozen in dry ice. A total homogenate aliquot was kept for Western blot validation of synaptic enrichment.

Electron microscopy

Synaptosome and synaptoneurosome pellets were fixed in 4% Paraformaldehyde, 2.5% Glutaraldehyde, 0.2% Picric acid in 0.1 M phosphate buffer, pH 7.4 for 2 h at room temperature plus overnight at 4°C, post-fixed in 1% Osmium tetroxide for 30 min, washed in PB, boiled distilled water, and 50% ethanol, incubated in 1% uranyl acetate in 70% ethanol, dehydrated through 15 min steps in a graded series of ethanol then propylene oxide, 50% propylene oxide/50% Durcupan resin, then 100% Durcupan resin overnight in a Leica EM TP processor. Samples were baked in Durcupan resin in agar capsules overnight at 60°C, cut on an Ultracut microtome (Leica) with a Histo Jumbo diamond knife (Diatome) into 70 nm sections which were mounted on nickel mesh grids. Electron micrographs were captured on a Zeiss Gemini 360 scanning electron microscope with an annular STEM detector.

Protein extraction

For protein extraction, samples we diluted 5-fold with Tris-HCl buffer pH 7.6 (100 mM Tris-HCl, 4% SDS, and Protease inhibitor cocktail EDTA-free [ThermoFisher Scientific, 78447]), followed by centrifuging for 25 min at 13,3000 RPM at 4°C. Then, the supernatant was collected in fresh tubes and heated at 70°C for 10 min. Samples were kept at −80°C for long-term storage.

Micro BCA

Micro BCA Protein Assay kit (ThermoFisher Scientific, 23235) was used to quantify protein levels, following manufacturer's instructions. Briefly, 1 μL of sample was added in 1 mL of working solution and heated at 60°C for 1 h. Working solution was made fresh right before required, consisting of 50% Buffer A, 48% Buffer B and 2% Buffer C, all provided in kit. Albumin was provided in the kit for determining a standard curve of the following concentration: 2 μg/mL, 4 μg/mL, 6 μg/mL, 8 μg/mL, 10 μg/mL, and 20 μg/mL. Absorbance values were obtained using spectrophotometry at 562 nm with working solution used as the blank value to calibrate the machine.

Western blot

After protein extraction, each sample was made-up to 20 μg of protein as calculated by the micro BCA (protein extract diluted in deionised water) and diluted in half with Laemmli buffer (2x stock) (S3401-10VL). In each well, 15 μL of sample were loaded in 4–12% Bis-Tris gels (ThermoFisher Scientific, NP0323BOX). Each gel was run with 5 μL of molecular weight marker (Licor, 928–40000) in the first well. Gels were washed with de-ionised water and diluted NuPAGE buffer (ThermoFisher Scientific, NP0002) (20x stock) to remove bits of broken gel and residual acrylamide. Western blot chambers were filled with diluted NuPAGE buffer (600 mL per chamber), making sure each chamber compartment was locked securely and there was no leaking. Gels were run at 80 V for 5 min, 100 V for 1.5 h and 120 V for 30 min. Then, using a scraper tool, gels were cracked open from their plastic casing and soaked in 20% ethanol for 10 min prior to transferring using the iBlot 2 Dry Blotting System (IB21001). Pre-packed transfer stacks containing a PVDF membrane (ThermoFisher Scientific, IB24002) were assembled as per manufacturers recommendations, and samples were transferred for 8.5 min at 25 V. After transferring, the PVDF membranes were stained for 5 min with Ponceau S in 5% acetic acid (P7170-1L), and washed 3 times with 5% acetic acid to stain for total protein. Ponceau S stained membranes were scanned and analyzed on FIJI to obtain a measurement of total protein per sample. Ponceau S was washed out with PBS and the PVDF membranes were blocked using Odyssey blocking buffer (Licor, 927–40000) for 30 min, following overnight incubation with primary antibodies made up in Odyssey block with 0.1% Tween 20, at 4°C with gentle shaking. The following primary antibodies were used: Synaptophysin (mouse, Abcam ab8049, 1:1000), PSD-95 (rabbit, Cell signaling D27E11, 1:1000), Histone (rabbit, Abcam ab1791, 1:1000), and MFG-E8 (sheep, R&D Systems AF2767, 1:500). The next day, membranes were washed 6 times with PBS-0.1% Tween 20, and incubated with the following LI-COR secondary antibodies for 30 min: IRDye 680RD Donkey anti-Mouse IgG, highly cross-adsorbed (LI-COR, 925–68072, 1:5000) and IRDye 800CW Donkey anti-Rabbit IgG, highly cross-adsorbed (LI-COR, 925–32213, 1:5000). All gels were imaged using the same exposure times and intensities using an LI-COR Scanner. The images were then uploaded on ImageStudio for analysis. For each band, the same size box was used to ensure all samples are measured equally. Each sample was normalized to its corresponding value of total protein.

Dot blots

Equal protein concentrations of protein-extracted synaptoneurosome samples were applied on nitrocellulose membrane using a vacuum dot blotter (CSL-D48, Cleaver Scientific). Membranes were air-dried and blocked with Odyssey blocking buffer (LI-COR, 927–40000) for 30 min, followed by incubation with an anti-MFG-E8 antibody (sheep, R&D Systems AF2767, 1:500) and rabbit anti-goat HRP-tagged secondary antibody (Abcam ab6741, 1:5000). Membranes were processed with SuperSignal West Dura Extended Duration Substrate (ThermoFisher Scientific, 34075) for 5 min and imaged on an LI-COR Scanner under chemiluminescence settings. Total protein was measured with Revert 700 (LI-COR, 926–11021). For each blot, the same size box was used to

ensure all samples are measured equally. Each sample was normalized to its corresponding value of total protein. Mouse hippocampus was used as a negative control and human milk was used as a positive control.

Synaptoneurosome and synaptosome labeling with pHrodo Red-SE

First, pHrodo Red-SE (ThermoFisher Scientific, P36600) was diluted with DMSO according to manufacturer's instructions to reach a concentration of 10 mM. Synaptoneurosome and synaptosome pellets were resuspended in 100 mM sodium carbonate buffer pH 9, adapted from a previously described protocol.[28] Synaptoneurosomes were tagged with pHrodo Red-SE based on protein concentration, roughly at 4 mg/mL, as calculated by the micro BCA, and incubated at room temperature under gentle shaking for 1 h, covered in foil. Samples were centrifuged at 13,000 RPM for 10 min to obtain the labeled synaptoneurosome pellet, followed by 3 rounds of washing with PBS and centrifugation to wash out any unbound dye. The pHrodo-labeled synaptoneurosome pellets were then resuspended in 5% DMSO-PBS and aliquoted for storage at −80°C. Aliquots of pooled AD and NDC samples were prepared fresh.

IncuCyte phagocytosis assay on astrocytes

Due to the long image-acquisition times acquired for astrocyte phagocytosis assays, these experiments were performed in an IncuCyte S3 live-imaging system that is housed in a tissue-cultured incubator. Phase images were taken prior to synaptoneurosome addition to normalize ingestion for the confluency of astrocytes per well. Astrocytes were imaged every 2 h with the 20X objective for up to 48 h on phase contrast and red channel to pick up the pHrodo-Red signal. To determine the amount of phagocytosis, the amount of pHrodo-Red was normalized to the respective phase contrast of the well at time = 0.

MFG-E8 and integrin (α5β5) antibody blocking *in vitro*

A recombinant mouse anti-human MFG-E8 blocking antibody (Mc3) (low endotoxin) was purchased by Creative Biolabs (FAMAB-0225CQ-LowE), along with an isotype control mouse IgG1 (MOB-065CQ). Both antibodies were used at a concentration of 10 μg/mL (as suggested by the supplier). Integrin α5β5 was blocked at a concentration of 2.5 μg/mL (R&D Systems, MAB2528). Synaptoneurosomes were incubated with the anti-MFG-E8 and IgG1 antibodies and cells were incubated with the anti-α5β5 and anti-IgG1 antibodies for 4 h at 37°C prior to challenging *in vitro*. Glial cells were blocked with Fc block (human: Biolegend, 422302) (mouse: Biolegend: 101302) at a dilution of 1:1000 for 30 min prior to synaptoneurosome challenging.

Degradation assay

Primary mouse and human microglia were incubated with 2.5 μL of pHrodo-tagged synaptoneurosome preparations from control and AD brains and were imaged for 2 h in IncuCyte S3 every 15 min. The media was then aspirated and cells were washed with warm fresh media twice, before returning them to IncuCyte to resume imaging for another 2 days, imaging every 2 h. Astrocytes were assessed similar by were imaged every 2 h for 48 h prior to washing. K777 (Adipogen, AG-CR1-0158) was added at a concentration of 1 μM to block cathepsins and prevent degradation as control.

Immunofluorescence of cultured cells

Plated cells were fixed for 10 min with 4% PFA. Subsequently cells were washed with PBS (pH 7.4) and incubated with 0.1% Triton X-100 for 30 min. After washing, blocking solution was applied for 1 h (10% NDS, 3% Bovine serum albumin [A8806-5G], 0.1% Triton X-100, 0.1% Tween 20 in PBS). Primary antibodies were diluted in blocking solution and used in the same concentrations as in the human paraffin tissue, and left overnight at 4°C. Cells were washed 3 times with PBS and secondary antibodies were applied for 30 min with DAPI (1 μg/mL). Secondaries and DAPI were washed, and cells were topped with PBS and kept in the fridge. Images were taken with 10x and 20x objectives using a Zeiss Axio Observer Z1 microscope.

QUANTIFICATION AND STATISTICAL ANALYSIS

Statistical analyses were performed in R Studio[54] using R version 4.1.2. The code for statistical analysis can be found on the Edinburgh Datashare repository https://datashare.ed.ac.uk/handle/10283/3076. For human postmortem studies, differences in demographics between groups were tested with analysis of variance for continuous variables (age, PMI|) and with Fisher's tests for factor data (*APOE* genotype, gender). To investigate differences between groups, linear mixed effects models were used with case or culture replicate as a random effect to account for multiple measurements from each case in order to avoid pseudoreplication of data while maximising statistical power.[55] Fixed effects in the models were disease status (AD or control), Brain region, *APOE4* status, gender, age and plaque proximity as relevant. Human postmortem glial colocalization with synapse data were normalized by dividing the volume of colocalized stains by the total volume of glial stain in each stack in the "normalized" data in Figure S1. Full statistical details including data spreadsheets, statistical models, and results can be found on the Edinburgh Datashare repository https://datashare.ed.ac.uk/handle/10283/3076.

Cell Reports Medicine, Volume *4*

Supplemental information

Human astrocytes and microglia show augmented ingestion of synapses in Alzheimer's disease via MFG-E8

Makis Tzioras, Michael J.D. Daniels, Caitlin Davies, Paul Baxter, Declan King, Sean McKay, Balazs Varga, Karla Popovic, Madison Hernandez, Anna J. Stevenson, Jack Barrington, Elizabeth Drinkwater, Julia Borella, Rebecca K. Holloway, Jane Tulloch, Jonathan Moss, Clare Latta, Jothy Kandasamy, Drahoslav Sokol, Colin Smith, Veronique E. Miron, Ragnhildur Thóra Káradóttir, Giles E. Hardingham, Christopher M. Henstridge, Paul M. Brennan, Barry W. McColl, and Tara L. Spires-Jones

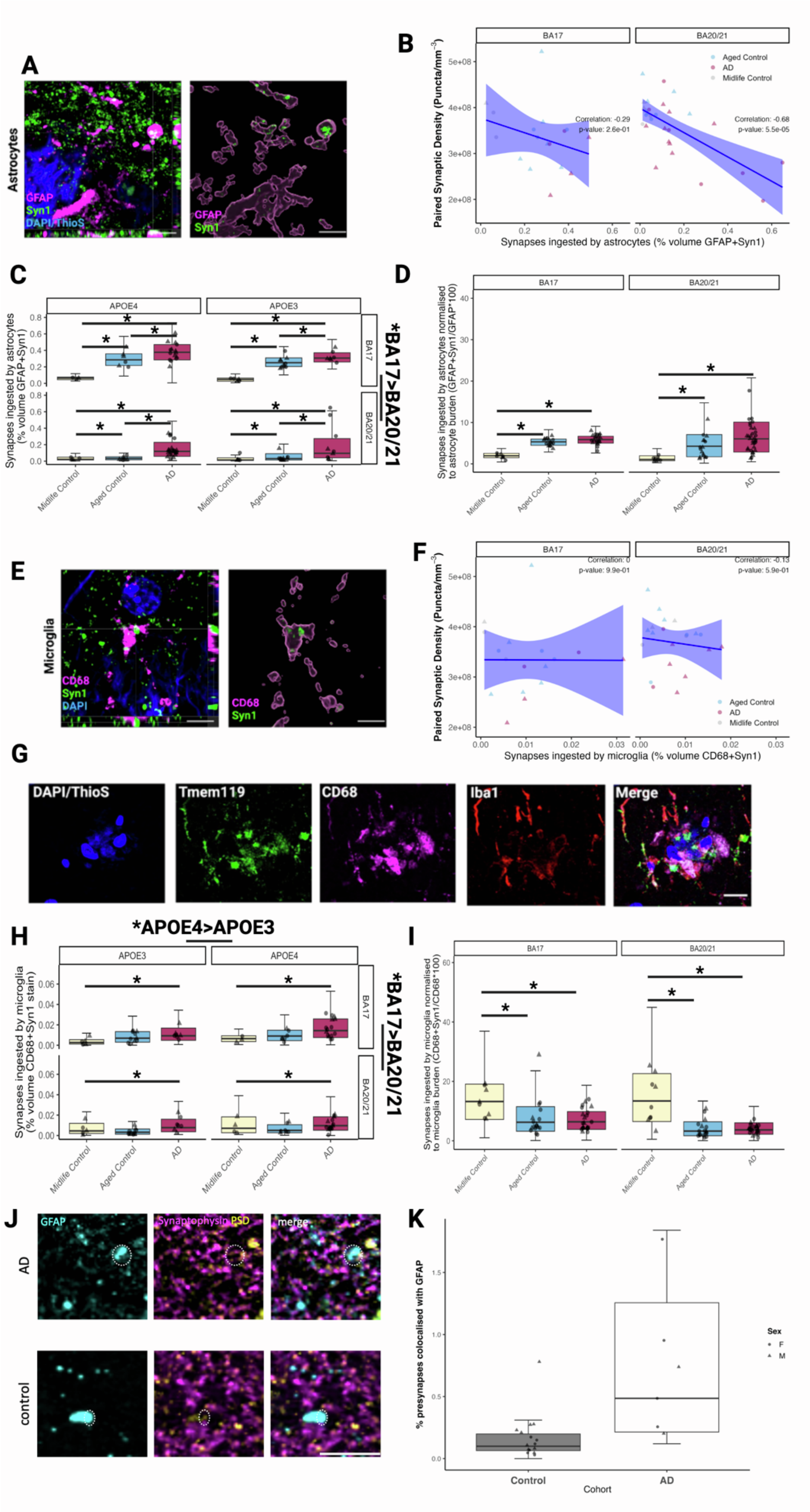

Figure S1. Supplementary statistics and validation microscopy, Related to Figures 1-3. (A) Super-resolution confocal using an Airyscan microscope confirm synaptic engulfment by astrocytes in AD (orthogonal view left , 3D reconstruction right). Scale bar represents 5µm. (B) Correlation analysis shows a significant negative correlation between

synapse density determined with array tomography and synaptic colocalization with astrocytes in BA20/21. (C) There is an effect of *APOE* genotype with *APOE4* carriers having more astrocyte engulfment than *APOE3* carriers. Also there is more engulfment in BA17 compared to BA20/21. (D) When normalized to GFAP burden, synaptic colocalization with GFAP was increased in AD compared to mid-life controls and increased in healthy ageing compared to mid-life controls. (E) Super-resolution confocal using an Airyscan microscope confirm synaptic engulfment by microglia in AD (orthogonal view left , 3D reconstruction right). Scale bar represents 5µm. (F) Correlation analysis shows no significant correlations between synapse density determined with array tomography and synaptic colocalization with microglia in either brain region. (G) CD68 staining was confirmed to colocalise with Tmem119 and Iba1. Scale bar represents 20µm. (H) There is an effect of *APOE* genotype with *APOE4* carriers having more astrocyte engulfment than *APOE3* carriers. Also there is more engulfment in BA17 compared to BA20/21. (I) When normalized to CD68 burden, the synaptic ingestion by microglia was no longer higher in AD cases compared to controls indicating the increase in AD in microglial ingestion of synapses is driven by microgliosis. (J) Array tomography imaging of GFAP and synaptic proteins confirms synaptic ingestion by astrocytes. Scale bar represents 10µm. (K) There is significantly more synaptic ingestion by astrocytes in AD than control when measured with array tomography (ANOVA after linear mixed effects model, F[1,16]=12.11, p=0.003).

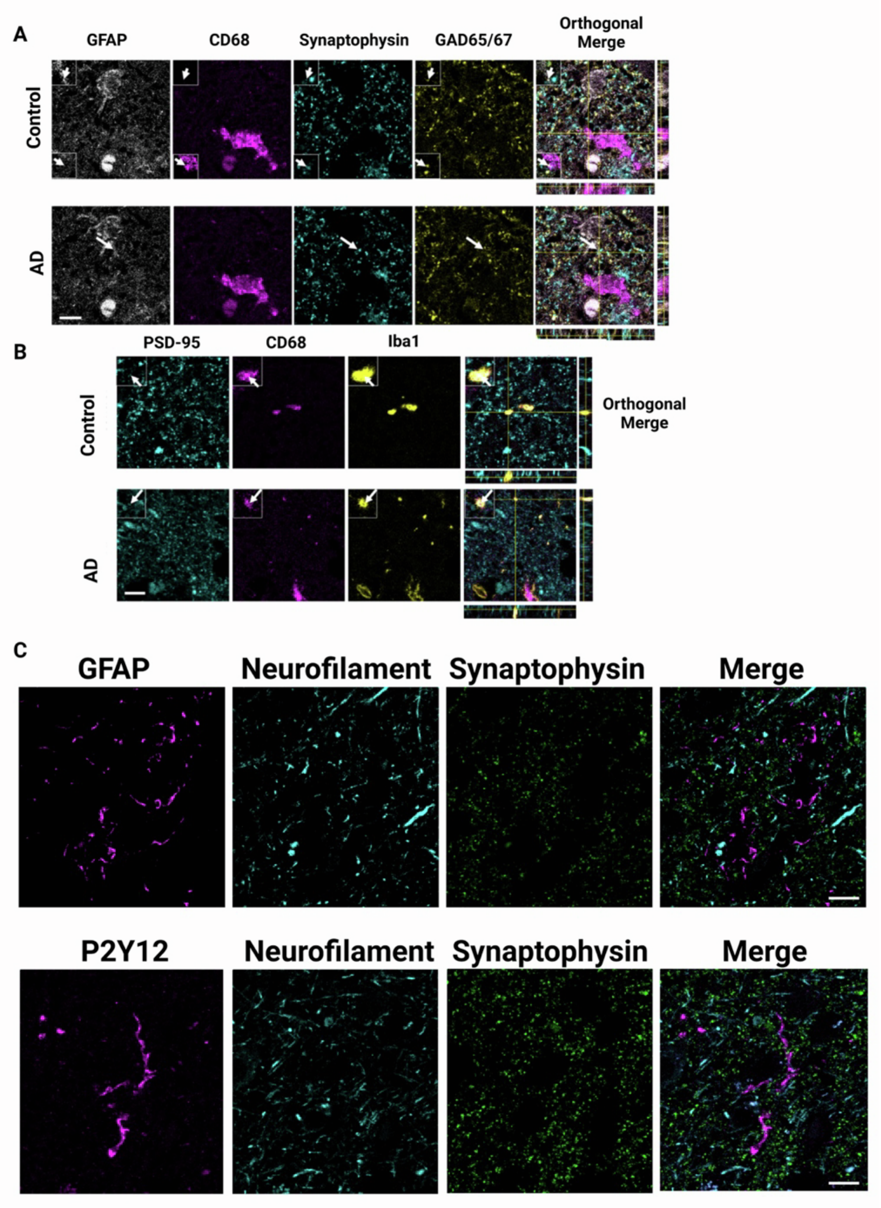

Figure S2. Glial co-staining with multiple synaptic markers. Related to Figures 1-3.
(A) Confocal images of staining with GFAP, CD68, synaptophysin, and GAD65/67 shows inhibitory synaptic protein inside astrocytes and microglia (arrows). (B) Excitatory postsynaptic protein PSD95 is also observed in CD68 and Iba1double positive microglia (arrows, B). (C) Staining with axonal neurofilament (cyan) alongside GFAP or P2Y12 (magenta) and synaptophysin (green) did not show substantial colocalization between astrocytes or microglia and axonal neurofilament. Scale bars represent 10mm. Insets are 5 x 5 mm.

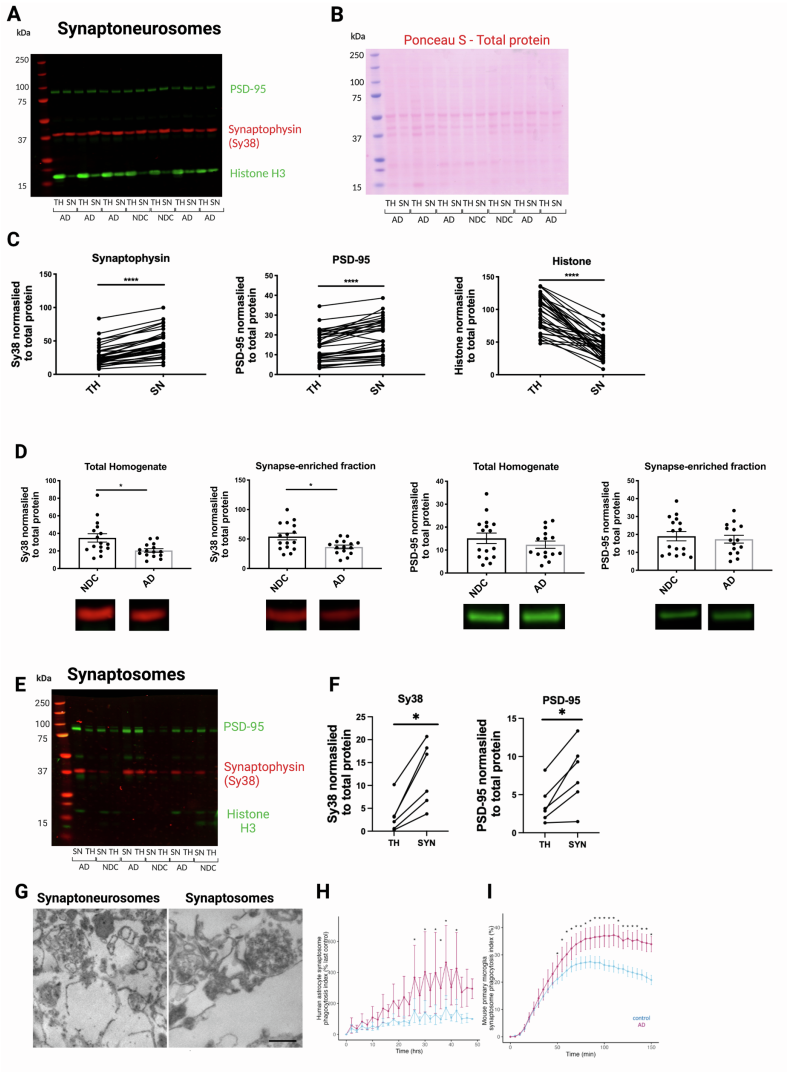

Figure S3. Validation of synaptoneurosome and synaptosome preparations. Related to Figures 4-6. (A) Representative image of full-length Western blot, indicating whether a sample is from total homogenate (TH) or synaptoneurosomes (SN), and their corresponding disease status. Bands were quantified on Image Studio, and normalised to total protein, quantified by Ponceau S. (B) Ponceau S staining for total protein from gel shown in A (NDC=no disease control, AD=Alzheimer's disease). (C) Significantly increased protein levels of the pre- and post-synaptic markers synaptophysin (Sy38) and PSD-95, respectively, as well as decreased protein levels of histone (H3), indicating exclusion of non-synaptic material (Wilcoxon matched-pairs signed rank test, ****p<0.0001, n=31). Lines link the two different preparations from the same case. (D) Decreased levels of synaptophysin (Sy38) in the total homogenate (Mann-Whitney test, p=0.0106) and synaptoneurosome fraction (unpaired Student's t-test, p=0.0121) of AD cases (n=15), compared to age-matched NDC cases (n=16), as detected by Western blot. PSD-95 protein levels were not different between AD and NDC groups in total homogenate (unpaired Student's t-test, p=0.332), nor in the synaptoneurosome fraction (unpaired Student's t-test, p=0.627). (E) Representative image of full-length Western blot for synaptophysin, PSD-95 and histone H3, indicating whether a sample is from total homogenate (TH) or synaptoneurosomes (SN), and their corresponding disease status. Bands were quantified on Image Studio, and normalised to total protein, quantified by Ponceau S. (F) Significantly increased protein levels of the pre- and post-synaptic markers synaptophysin (Sy38) and PSD-95, respectively, (Wilcoxon matched-pairs signed rank test, *p<0.05, n=3). Lines link the two different preparations from the same case. (G) Synaptoneurosome and synaptosome pellets were embedded for electron microscopy confirming synaptic structures in both types of synaptic enrichment (scale bar 300 nm). (H) As seen with synaptoneurosomes, pHrodo tagged human synaptsomes from AD brain were phagocytosed more and faster by human astrocytes. (I) AD-derived human synapotosomes also ingested more than control by mouse microglia, similar to the synaptoneurosomes. For statistics, *p<0.05.

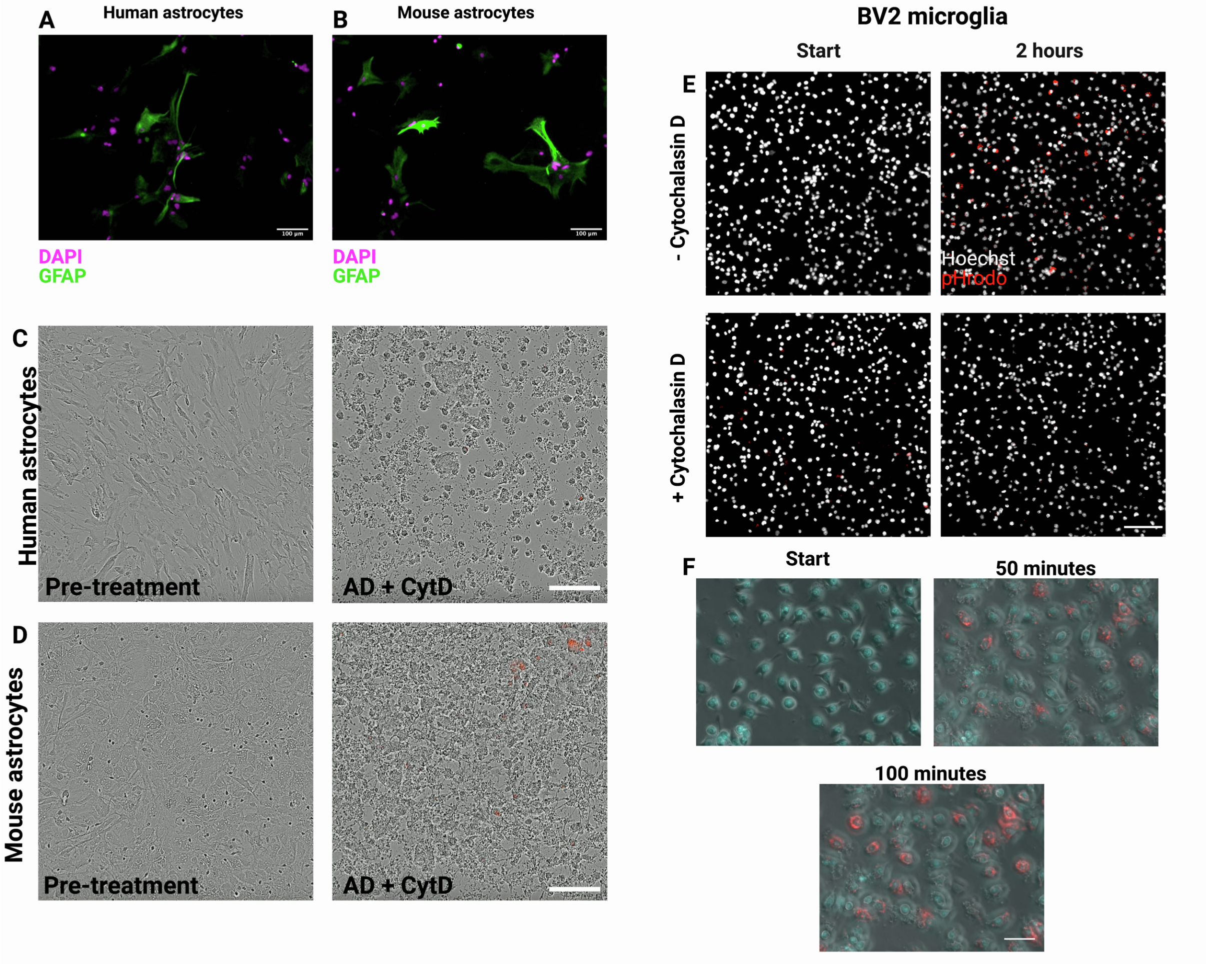

Figure S4. Validation of synaptoneurosome ingestion by astrocytes and microglia. Related to Figures 4-6. (A) Primary human astrocytes stained for GFAP (green) shows most cells express the marker, indicating a pure astrocytic culture. (B) Primary mouse astrocytes stained for GFAP (green) shows cells express the marker, although some cells do not. Scale bars represent 100µm. (C) Primary human astrocytes treated with cytochalasin D prior to phagocytosis assay, blocking phagocytosis. Scale bar 200µm. (D) Primary mouse astrocytes treated with cytochalasin D prior to phagocytosis assay, blocking phagocytosis. Scale bar 200µm. (E) Still images from live imaging of BV2 microglia (Hoechst-positive nuclei in grey) undergoing phagocytosis of human synaptoneurosomes tagged with pHrodo. Synaptoneurosomes can be seen in red as they enter the acidic phago-lysosomal compartment of the cell. (F) Cells treated with 10µM of Cytochalasin D 30 minutes prior to the experiment showed no phagocytosis. Scale bar represents 30µm. (G) Live imaging of BV2 microglia (phase with Hoechst-positive nuclei in cyan) undergoing phagocytosis of human synaptoneurosomes tagged with pHrodo (can be seen as small spheroids on phase contrast). Synaptoneurosomes become red once they enter the acidic phago-lysosomal compartment of the cell. Each panel represents an image 50 minutes apart. Scale bar represents 50µm.

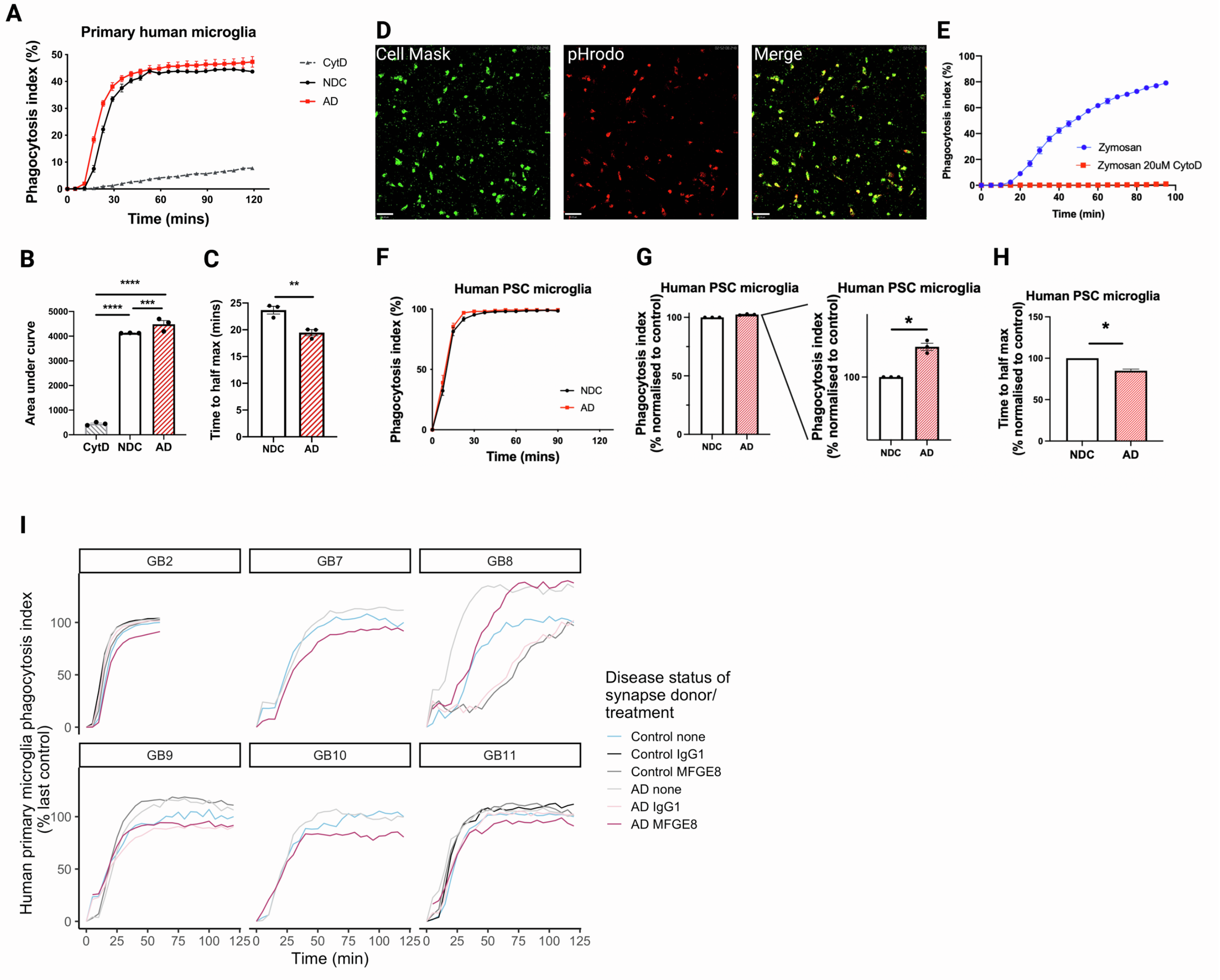

Figure S5. Increased phagocytosis of AD-derived synaptoneurosomes by human iPSC and primary microglia. Related to Figures 5 and 6. (A) Phagocytosis index of primary human microglia from an epilepsy case engulfing human synaptoneurosomes (n=1 human sample, replicated in 3 wells from an average of 9 images per well). CytD= cytochalasin D, NDC= non-demented control, AD= Alzheimer's disease. (B) Area under curve from A (one-way ANOVA with Tukey's multiple comparisons test, p=0.0004). (C) Time to half-maximum phagocytosis was calculated from the curve shown in A (unpaired Student's t-test, p=0.0098). For statistics, **p<0.01, ***p<0.001, ****p<0.0001. Data shown as mean ± SEM. (D) Representative images of human PSC derived microglia-like cells labelled with Cellmask Deep red (green), showing phagocytosed pHrodo labelled synaptoneurosomes (red) at the end of imaging time. Scalebar: 80µm. (E) Zymosan beads are readily phagocytosed by human PSC microglia in culture, and treatment of cytochalasin D (CytoD) is sufficient to completely abolish it. Data shown as mean ± SEM. (F) All cell lines showed phagocytosis of SNS particles, the responsiveness and dynamics of microglia to AD and control material uptake depends on the individual cell lines. (G) Total amount of labelled particles as measured by the area under the curve was increased in PSC derived microglia presented with synaptoneurosomes from AD brain compared to NDC (one sample t-test, p=0.0142, hypothetical value=100). (H) PSC derived microglia-like cells phagocytosed AD SNS particles faster compared to NDC particles measured by the half max time (one sample t-test, p=0.016, hypothetical value=100). In all conditions, n=3 indicating lines from separate iPSC donors. For statistics, *p<0.05, and data shown as mean ± SEM). CytD= cytochalasin D, NDC= no disease control, AD= Alzheimer's disease. (I) The small surgical samples from which microglia were derived were not always sufficient to test all conditions. From our 11 donors, all had AD vs control synapse conditions, two had these and in addition AD synapses treated with MFG-E8 antibody, and 4 included all conditions (control and AD synapses with anti-MFG-E8, IgG1 control, or no treatment). In all 6 cases with MFG-E8 antibody pre-incubation, MFG-E8 antibody pre-treatment reduced AD synaptic phagocytosis compared to AD without antibody pre-treatment. In the 4 cases with IgG control, 2 had clear reduction in phagocytosis with MFGE8 compared to control IgG (GB11, GB2), one had no difference between MFGE8 and IgG (GB9), and one had increased phagocytosis with MFGE8 compared to IgG (GB8). From these data we conclude that while there is not a clear rescue of phenotype specifically with the MFG-E8 antibody treatment as seen for astrocytes, the microglial data show "responders" and "non-responders" to this treatment compared to IgG1 and all cases had a clear reduction in phagocytosis with either IgG1 or MFGE-8 treatment indicating that IgG on synapses may be an opsonin recognized by microglia .

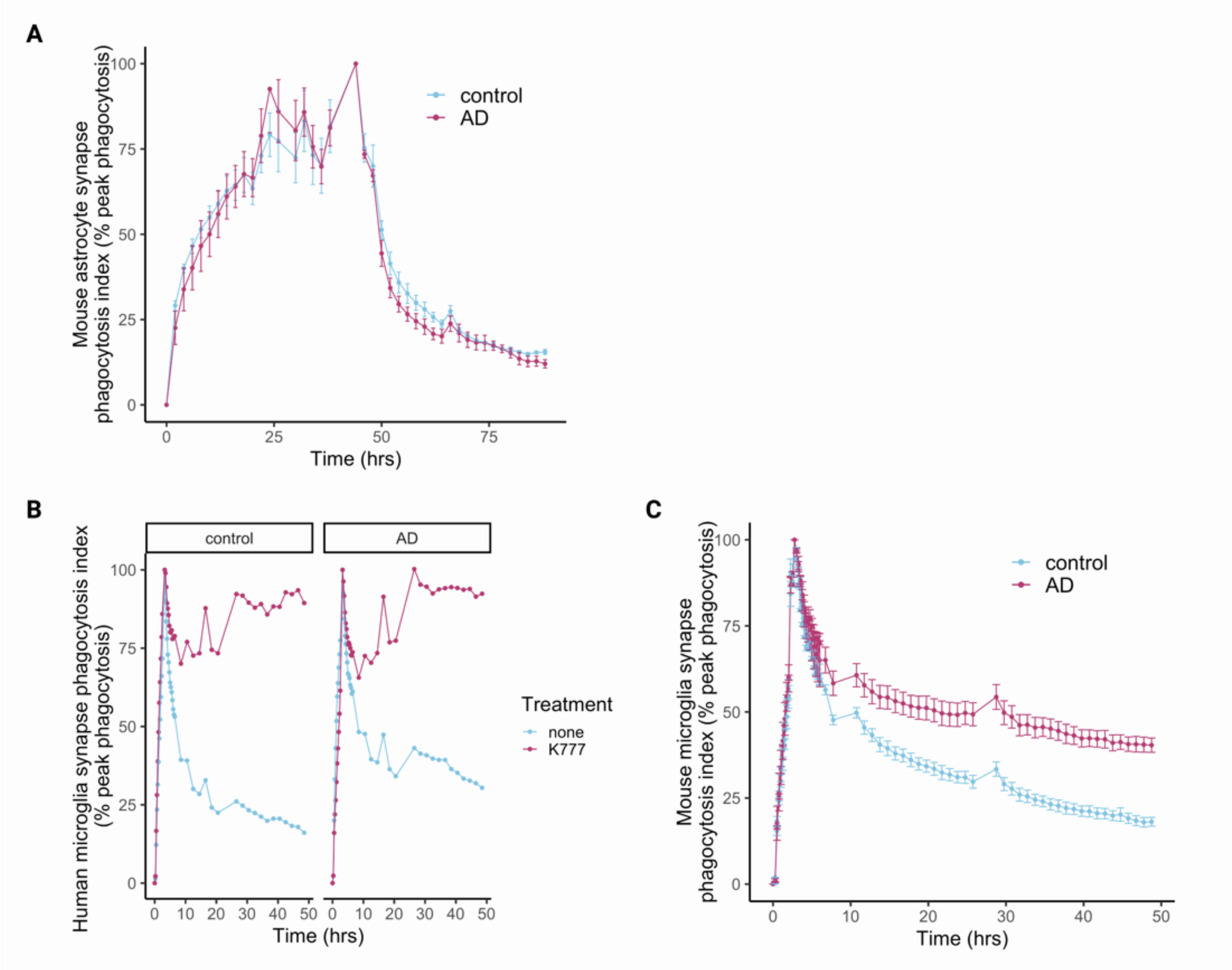

Figure S6. Degradation assays of mouse and human primary microglia, Related to Figures 5 and 6. (A) Degradation assay in primary mouse astrocytes (n=4 replicates) of human control and Alzheimer's disease (AD) synaptoneurosomes shows no differences in degradation throughout the assay. (B) Degradation assay in primary human microglia (n=1, GB12) of human control and Alzheimer's disease (AD) synaptoneurosomes shows no differences in degradation in the 2 hours of the assay. Both control and AD synaptoneurosomes are efficiently degraded over time, which is blocked by the pan-cathepsin inhibitor K777. (C) Degradation assay in primary mouse microglia (n=4 adult mice) of human control and Alzheimer's disease (AD) synaptoneurosomes shows no differences in degradation in the first 2 hours of the assay. Both control and AD synaptoneurosomes are efficiently degraded over time.

BBN/ CaseID	SD number	Disease	APOE	Age (years)	Gender	PMI (hrs)	Braak Stage	Experiment
29693	SD002/17	Midlife control	APOE4	49	F	94	NA	IHC
24479	SD005/15	Midlife control	APOE4	46	F	76	NA	IHC
33613	SD014/18	Midlife control	APOE3	46	F	99	NA	IHC
28959	SD022/16	Midlife control	APOE3	39	M	86	NA	IHC
30169	SD022/17	Midlife control	APOE4	48	M	58	NA	IHC
28960	SD026/16	Midlife control	APOE3	37	F	126	NA	IHC
34244	SD036/18	Midlife control	APOE3	49	F	69	NA	IHC
24342	SD053/14	Midlife control	APOE3	33	M	47	NA	IHC
29906	SD013/17	Midlife control	APOE4	51	M	52	NA	IHC
1.28793	SD017/16	Aged Control	APOE3	79	F	72	2	IHC
14395	SD014/13	Aged Control	APOE3	74	F	41	NA	IHC + SNS
19686	SD063/13	Aged Control	APOE3	76	F	75	1	IHC + SNS
20122	SD003/14	Aged Control	APOE3	59	M	74	NA	IHC + SNS
22612	SD022/14	Aged Control	APOE3	61	M	70	NA	IHC + SNS
26495	SD024/15	Aged Control	APOE3	78	M	39	1	IHC + SNS
28402	SD051/15	Aged Control	APOE3	78	M	49	1	IHC + SNS
28406	SD001/16	Aged Control	APOE3	79	M	72	2	IHC + SNS
28797	SD025/16	Aged Control	APOE3	79	M	57	NA	IHC + SNS
29086	SD034/16	Aged Control	APOE3	79	F	68	NA	IHC + SNS
32577	SD002/18	Aged Control	APOE3	81	M	74	2	IHC
1.34131	SD029/18	Aged Control	APOE4	82	M	95	4	IHC
15809	SD029/13	Aged Control	APOE4	58	M	90	NA	IHC + SNS
16425	SD032/13	Aged Control	APOE4	61	M	99	NA	IHC + SNS
20593	SD006/14	Aged Control	APOE4	60	M	52	NA	IHC + SNS
22629	SD035/14	Aged Control	APOE4	59	F	53	NA	IHC + SNS
29082	SD031/16	Aged Control	APOE4	79	F	80	3	IHC + SNS
31495	SD043/17	Aged Control	APOE4	81	M	38	6	IHC + SNS
1.29081	SD030/16	AD	APOE3	90	F	110	2	IHC
1.30142	SD020/17	AD	APOE3	88	F	112	2	IHC
15258	SD026/13	AD	APOE3	65	M	80	6	IHC + SNS
19595	SD062/13	AD	APOE3	87	M	58	6	IHC + SNS
19994	SD002/14	AD	APOE3	87	F	89	6	IHC + SNS
24527	SD056/14	AD	APOE3	81	M	74	5	IHC
28410	SD005/16	AD	APOE3	62	F	109	6	IHC + SNS
28771	SD010/16	AD	APOE3	85	M	91	6	IHC

32929	SD012/18	AD	APOE3	87	F	83	4	IHC
1.265	SD039/15	AD	APOE4	81	M	83	6	IHC
1.26732	SD048/15	AD	APOE4	76	M	66	6	IHC + SNS
1.28796	SD021/16	AD	APOE4	60	F	54	6	IHC
1.29135	SD027/16	AD	APOE4	90	M	73	6	IHC
1.30883	SD034/17	AD	APOE4	61	F	69	6	IHC
1.30973	SD039/17	AD	APOE4	89	F	96	6	IHC
1.31499	SD044/17	AD	APOE4	85	M	78	6	IHC
1.33636	SD017/18	AD	APOE4	93	M	43	2	IHC
1.33698	SD022/18	AD	APOE4	90	F	76	NA	IHC
10591	SD003/13	AD	APOE4	76	M	76	6	IHC + SNS
15256	SD034/13	AD	APOE4	60	M	28	5	IHC
15810	SD018/13	AD	APOE4	73	F	96	6	IHC
15811	SD021/13	AD	APOE4	81	F	41	6	IHC
19690	SD064/13	AD	APOE4	57	M	58	6	IHC + SNS
20995	SD019/14	AD	APOE4	60	M	86	6	IHC + SNS
23394	SD038/14	AD	APOE4	88	F	59	5	IHC + SNS
24322	SD049/14	AD	APOE4	80	M	101	6	IHC + SNS
24526	SD055/14	AD	APOE4	79	M	65	6	IHC
24668	SD058/14	AD	APOE4	96	F	61	6	IHC
25739	SD014/15	AD	APOE4	85	F	45	6	IHC + SNS
26718	SD040/15	AD	APOE4	78	M	74	6	IHC + SNS
29521	SD035/16	AD	APOE4	95	M	96	6	IHC + SNS
29695	SD004/17	AD	APOE4	86	M	72	6	IHC + SNS

Table S1. Human cases used in the study. (NA=not available, IHC = immunohistochemistry, SNS = synaptoneurosome)

CaseID	Age	Gender	Brain Region resected
GB1	55	M	parietal lobe
GB2	50	F	temporal lobe
GB3	50	M	frontal lobe
GB5	50	F	parietal lobe
GB6	60	F	parietal lobe
GB7	60	F	frontal lobe
GB8	60	F	frontal lobe
GB9	50	F	frontal lobe
GB10	60	M	frontal lobe
GB11	70	M	frontal lobe
GB12	71	M	parietal-occipital lobe

Table S2. Data from human donors from glioblastoma surgeries, Related to figures 5 and 6.
